# Longitudinal refractive changes following orbital decompression in thyroid eye disease: dominant role of axial configuration and globe position with contributions from surgical technique and corneal biomechanics

**DOI:** 10.1186/s40662-026-00499-9

**Published:** 2026-07-22

**Authors:** Mingqian Yu, Jiaqi Tang, Yueyue Li, Yue Li, Gaojing Jing, Youhan Ao, Rui Ma, Feng Liu, Mei Ge, Qinghua Yang, Meiluo Zhang, Mengyu Peng, Bing Nie, Xinji Yang, Wei Wu, Liqiang Wang

**Affiliations:** 1 School of Medicine, Nankai University, Tianjin, 300071 China; 2 Senior Department of Ophthalmology, 3rd Medical Center of Chinese PLA General Hospital, Beijing, 100080 China; 3 School of Clinical Medicine, Shandong Second Medical University, Weifang, 261000 China; 4 Department of Endocrinology, The First Hospital of Lanzhou University, the First Clinical Medical College, Lanzhou University, Lanzhou, 730000 China

**Keywords:** Thyroid eye disease, Orbital decompression, Longitudinal refractive change, Measured axial length, Corneal biomechanics and tomography, Generalized estimating equations

## Abstract

**Background:**

The purpose of this study was to characterize the longitudinal refractive outcomes of orbital decompression in thyroid eye disease (TED), compare refractive trajectories across decompression strategies, and identify the anatomical correlates and baseline predictors of postoperative refractive changes.

**Methods:**

This retrospective longitudinal study included 57 eyes of 46 patients with moderate-to-severe TED who underwent orbital decompression. Comprehensive ophthalmic evaluations including eyelid position parameters, refractive and axial measurements, and corneal biomechanical and tomographic assessments were performed preoperatively and during postoperative follow-up. Postoperative observations were analyzed using generalized estimating equations (GEE) models with restricted cubic splines, with time treated as a continuous variable to allow flexible modeling of temporal patterns. Multivariate GEE models were used to compare decompression strategies, evaluate associations between postoperative ocular biometric changes and postoperative refractive changes, and identify baseline predictors of clinically significant postoperative myopic drift.

**Results:**

Orbital decompression was associated with a biphasic pattern of refractive change characterized by an initial myopic shift, followed by partial hyperopic recovery over time (*P* < 0.001), with the greatest myopic shift occurring at approximately 2–3 months postoperatively. The refractive trajectories differed significantly across decompression strategies. Compared with one-wall decompression, two-wall decompression demonstrated a greater magnitude and more rapid progression of refractive change, as well as an increased risk of clinically significant myopic drift, independent of decompression extent. Standardized GEE analyses identified postoperative changes in measured axial length as the strongest correlate of postoperative refractive change (*P* < 0.001). Additional associations were observed for changes in exophthalmos, eyelid position, corneal biomechanics, and corneal tomographic parameters. Several baseline ocular biometric parameters were independently associated with susceptibility to clinically significant postoperative myopic drift.

**Conclusions:**

Orbital decompression induces dynamic postoperative refractive changes that vary according to decompression strategy and individual ocular characteristics. Postoperative refractive changes appeared to peak at approximately 2–3 months and partially recovered thereafter, with refractive status gradually approaching relative stabilization over time, suggesting that definitive refractive correction may be better deferred until postoperative stabilization. These findings provide clinically relevant insights into postoperative refractive behavior and may support individualized surgical planning and perioperative management for TED.

**Supplementary Information:**

The online version contains supplementary material available at 10.1186/s40662-026-00499-9.

## Background

Thyroid eye disease (TED) is an autoimmune orbital disorder characterized by the inflammatory expansion of extraocular muscles and orbital adipose tissue that results in proptosis, diplopia, and visual dysfunction. For patients with inactive, moderate-to-severe TED, orbital decompression remains the standard surgical intervention to reduce proptosis and orbital congestion [1–3]. Different surgical approaches yield distinct decompression effects depending on the extent of bone and fat removal, thereby inducing heterogeneous alterations in orbital geometry and the biomechanical environment of the globe [3, 4].

Beyond its established anatomical benefits, orbital decompression has been increasingly recognized to induce postoperative refractive changes. Even modest refractive shifts may be clinically meaningful, particularly in patients with high visual demands or those undergoing cataract or corneal refractive surgery [5, 6]. However, the existing evidence on refractive outcomes remains limited and inconsistent. Early studies reported notable postoperative myopic shifts, with magnitudes of approximately 1.00–2.50 diopters (D) [7], and a larger retrospective cohort demonstrated significant early myopic changes in both spherical diopter (DS) and spherical equivalent (SE) within 2 months after surgery [8]. In contrast, other studies have reported minimal or negligible changes in refractive status following orbital decompression [9–11]. Many previous investigations have summarized postoperative outcomes at discrete follow-up time points, which may not fully capture the dynamic and evolving nature of refractive changes over time.

These discrepancies suggest that postoperative refractive changes are context-dependent, time-varying, and influenced by multiple interacting factors. Among these, variations in the surgical approach may represent an important but insufficiently characterized source of heterogeneity. Quantitative imaging studies have demonstrated that balanced orbital decompression produces greater orbital volume expansion than one-wall decompression, accompanied by more pronounced posterior and horizontal globe displacement and greater reduction in proptosis [12]. Consistently, prospective studies have reported greater postoperative alterations in ocular motility following multi-wall decompression, indicating more extensive biomechanical perturbation of the extraocular muscle system [13]. Such positional and mechanical changes may propagate to the globe and anterior segment, potentially altering optical geometry and contributing to refractive change [8, 14]. However, whether these technique-dependent biomechanical differences translate into distinct longitudinal refractive trajectories has not been systematically investigated.

In addition to surgical factors, inter-individual variability in postoperative refractive outcomes suggests an important role of intrinsic ocular characteristics. Baseline ocular biometric features—including corneal curvature, pachymetric distribution, and anterior segment configuration—may modulate biomechanical responses to decompression. Evidence from studies using Corvis ST supports this concept, demonstrating that baseline SE is associated with postoperative changes in A2 length, defined as the length of the flattened corneal segment at the second applanation, which has been regarded as a surrogate marker of corneal resistance to deformation, whereas baseline Hertel exophthalmometry values are associated with alterations in A2 time [15]. These findings suggest that preoperative ocular structure and biomechanics may act as individualized “biomechanical set points,” shaping susceptibility to postoperative refractive change and supporting the potential value of comprehensive preoperative biometric profiling for individualized risk stratification and surgical planning.

Several mechanisms have been proposed to explain postoperative refractive changes following orbital decompression, including alterations in axial length (AL) [8], redistribution of extraocular muscle tension [16], and changes in corneal biomechanics and tomography [14, 15]. Orbital decompression fundamentally alters the biomechanical environment of the globe, which may affect corneal deformation behavior and anterior segment morphology [14]. These structural and biomechanical changes may collectively contribute to postoperative refractive variability [17]. Advances in imaging modalities, such as Corvis ST and Pentacam, have enabled comprehensive and dynamic assessment of corneal biomechanics and tomography in vivo. Integration of these measurements with longitudinal refractive and axial data may facilitate a more precise delineation of their relative contributions to refractive changes following orbital decompression.

Therefore, we conducted a retrospective longitudinal observational study of patients with TED who underwent orbital decompression with repeated postoperative measurements. Using generalized estimating equations (GEE)-based models with continuous time specification, we aimed to: (1) characterize the temporal trajectories of postoperative refractive change; (2) compare the magnitude, rate, and risk of postoperative myopic drift across surgical approaches, applying a causal framework to distinguish overall clinical effects from strategy-specific effects independent of decompression extent; (3) quantify the hierarchical and exposure–response relationships among postoperative axial, eyelid-related, corneal biomechanical, and morphological changes and refractive outcomes; and (4) identify baseline ocular biometric predictors of clinically significant postoperative myopic drift. By integrating these complementary analytical dimensions within a unified analytical framework, this study sought to provide mechanistic insights into refractive behavior following orbital decompression and inform risk stratification and individualized surgical planning.

## Methods

### Ethics statement

This retrospective longitudinal observational study was conducted as a secondary analysis of data derived from a prospective institutional cohort of patients with TED (project number KY2024–002). Comprehensive ophthalmic examinations and postoperative follow-up data were routinely collected as part of standard clinical care. No additional interventions were performed for the present analysis. The parent study protocol was approved by the Medical Ethics Committee of 3rd Medical Center of Chinese People’s Liberation Army General Hospital. This secondary analysis was conducted in accordance with the tenets of the Declaration of Helsinki and was within the scope of the approved protocol. Written informed consent was obtained from all participants before their inclusion in the institutional cohort.

### Participant enrollment

Consecutive patients with moderate-to-severe TED who underwent orbital decompression at the Senior Department of Ophthalmology, 3rd Medical Center of Chinese People’s Liberation Army General Hospital, between May 2024 and February 2025 were retrospectively identified from the institutional cohort database.

Eligibility was based on the following criteria: (1) diagnosis of TED according to Bartley’s criteria [18] with moderate-to-severe disease defined in accordance with the 2021 European Group on Graves’ Orbitopathy guidelines (EUGOGO) [1]; and (2) presence of severe proptosis, diplopia, or other symptoms substantially affecting the appearance or quality of life [1, 3].

Orbital decompression surgery was performed according to the standard institutional clinical practice. The surgical exclusion criteria included: (1) abnormal thyroid function defined as serum free triiodothyronine (FT3), free thyroxine (FT4), or thyroid-stimulating hormone (TSH) concentrations outside the reference range within 6 months before surgery; (2) uncontrolled systemic diseases (such as hypertension, diabetes mellitus, renal disease, or cardiovascular disorders); (3) pregnancy or lactation; and (4) diagnosed psychiatric disorders, including mood, anxiety, or psychotic disorders.

Standardized preoperative examinations were retrieved for all included cases, and all measurements were completed within 1 month prior to surgery. Postoperative examination data were retrospectively collected from routine clinical follow-up records. In standard clinical practice, patients were generally advised to attend follow-up visits at approximately 1 and 3 months after surgery. However, the actual timing of visits varied across individuals. For descriptive visualization purposes only, postoperative observations were additionally summarized within two broad follow-up windows reflecting routine clinical practice: an earlier postoperative window (< 2 months after surgery) and a later postoperative window (≥ 2 months after surgery). These descriptive windows were not incorporated into the primary inferential framework. All postoperative examinations were indexed relative to the date of surgery (time zero), which served as the reference point for all longitudinal analyses.

**Fig. 1:**
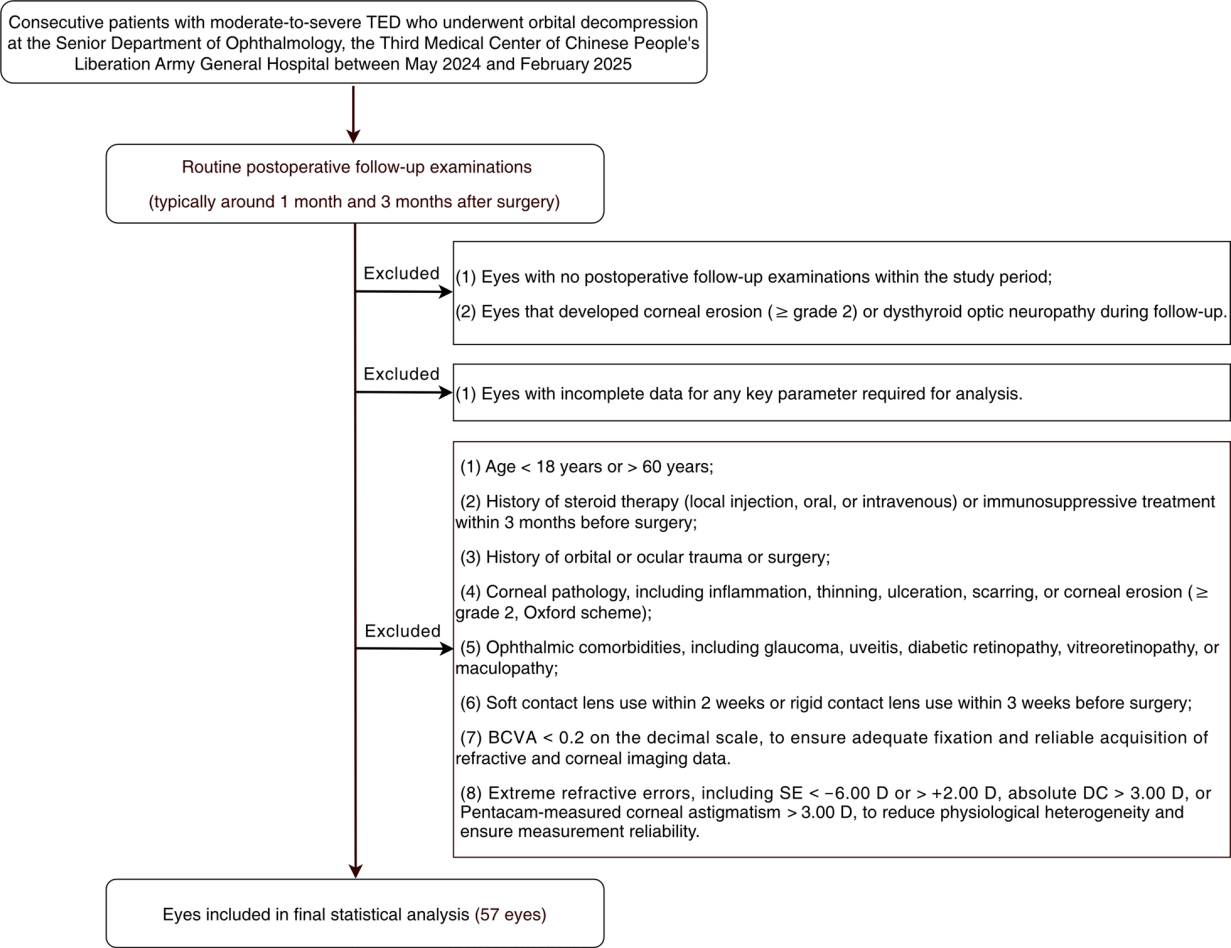
Study flowchart illustrating patient selection from an institutional cohort. Eligible eyes were identified from an ongoing institutional cohort of patients with TED after application of inclusion and exclusion criteria for this secondary analysis. BCVA, best-corrected visual acuity; DC, cylindrical diopter; SE, spherical equivalent; TED, thyroid eye disease

For analytical purposes, eyes with no postoperative follow-up examinations and those that developed corneal erosion (≥ grade 2) or dysthyroid optic neuropathy during follow-up were excluded first. Subsequently, eyes were excluded due to incomplete documentation of any key parameter required for analysis. Additional exclusions were applied based on a comprehensive review of clinical and ophthalmic data, as detailed in Fig. 1 and Supplementary Methods S1 in Additional File 1.

### Ocular examinations and surgical approaches

All demographic and clinical data, including preoperative and postoperative ocular measurements, were retrospectively retrieved from the electronic medical record system. Comprehensive ophthalmic data recorded in routine clinical practice included Clinical Activity Score (CAS); disease severity grading according to the EUGOGO guidelines; exophthalmos and eyelid position measurements; subjective and objective refraction; slit-lamp and fundus examinations; non-contact tonometry; optical biometry-based AL measurements; and corneal biomechanical and corneal tomographic assessments.

Disease activity was graded using the 7-item CAS system (CAS ≥ 3 indicating active disease) [19]. Exophthalmos was measured using Hertel exophthalmometry, with values > 16 mm indicating proptosis [20, 21]. Eyelid position was evaluated using palpebral fissure height (PFH) and margin reflex distance 1 (MRD1) measured in primary gaze under standardized conditions using a penlight and calibrated ruler. PFH was defined as the vertical distance between the upper and lower eyelid margins at the pupil center in primary gaze [22]. MRD1 was defined as the distance from the corneal light reflex to the upper eyelid margin in primary gaze, a widely used metric for quantifying upper eyelid position and retraction severity [23, 24].

Objective refraction was performed using a noncycloplegic autorefractor (ARK-1; Nidek Co., Ltd., Gamagori, Japan), followed by subjective refraction refinement. Consistent with previous studies, only autorefractor-derived objective refractive measurements were used for statistical analyses to ensure standardization and reproducibility [8, 9, 25–27]. Slit-lamp biomicroscopy and fundus examinations were performed using standard slit-lamp and fundus lens systems. Intraocular pressure (IOP) was measured using a non-contact tonometer (TX-20, Canon, Tokyo, Japan).

AL was measured using optical biometry (AL-Scan, Nidek Co., Ltd., Gamagori, Japan), which determines the optical distance from the corneal apex to the retinal pigment epithelium using low-coherence reflectometry [28–30]. As optical biometry captures the effective optical path length rather than the true anatomical length [31], AL in this study was interpreted as an optical surrogate rather than an anatomical measure. Postoperative changes in AL (ΔAL) may therefore reflect alterations in globe position, alignment, and overall optical geometry.

The corneal biomechanical properties were assessed using the Corvis ST system (Oculus Optikgeräte GmbH, Wetzlar, Germany; software version 1.6b2042). Recorded parameters included applanation-based indices, deformation amplitude metrics, curvature-related parameters, and composite biomechanical indices [32, 33]. The mean of three consecutive measurements that met the built-in quality specifications was used for analysis. Corneal tomography was performed using the Pentacam AXL system (Oculus Optikgeräte GmbH, Wetzlar, Germany; software version 1.29r12). The tomographic parameters included anterior and posterior keratometry (K), Belin/Ambrósio Enhanced Ectasia Display indices, corneal shape and irregularity metrics, pachymetry-derived indices, and anterior chamber measurements [34–36]. One high-quality scan rated “OK” according to the built-in quality specifications was accepted for analysis, as each scan is based on multiple Scheimpflug images collected during a single acquisition [37].

All measurements were conducted under standardized conditions in accordance with the protocols recommended by the manufacturers [37, 38]. All assessments were performed under non-cycloplegic conditions, consistent with routine clinical practice during adult postoperative follow-up. Detailed definitions of all biomechanical and tomographic parameters are provided in Additional File 2. For parameters measured using multiple devices, the central corneal thickness (CCT) obtained using Pentacam [CCT(p)] was adopted as the representative CCT parameter for subsequent analyses, given its widespread clinical use and integration with topographic and densitometric data [39, 40]. The IOP measured using Corvis ST [IOP(c)] was selected as the representative IOP index because it accounts for corneal biomechanics and minimizes thickness- and stiffness-related measurement artifacts [14, 41, 42].

Orbital decompression procedures were categorized as balanced medial–lateral wall decompression (BMLD), lateral wall decompression (LWD), and medial wall decompression (MWD), all combined with orbital fat removal. The surgical approach was selected based on clinical factors including proptosis severity, disease activity, orbital anatomy, cosmetic considerations, and surgeon experience [43, 44]. Detailed descriptions of the surgical procedures are provided in Supplementary Methods S2 of Additional File 1. For bilateral cases, surgeries were performed sequentially at intervals of approximately 1 month, and each eye was analyzed independently.

### Analytical framework and statistical methodology

#### Baseline characteristics and follow-up time

Baseline demographic and clinical characteristics were summarized for the overall cohort. Continuous variables were presented as mean ± standard deviation (SD) or median [interquartile range (IQR)], depending on their distribution as assessed by the Shapiro–Wilk test, while categorical variables were summarized as counts and percentages.

Follow-up time was defined as the interval (in months) between the date of surgery and each postoperative examination. All available postoperative measurements were retained for longitudinal analyses without restriction to predefined follow-up intervals, and follow-up time was treated as a continuous variable. The distribution of follow-up time was evaluated descriptively using a histogram and kernel density estimation, and normality was assessed using the Shapiro–Wilk test.

For descriptive visualization and clinically intuitive interpretation, postoperative observations were additionally summarized within two broad follow-up windows reflecting routine clinical follow-up patterns: an earlier postoperative window (< 2 months after surgery) and a later postoperative window (≥ 2 months after surgery). Within each follow-up window, preoperative and postoperative refractive and axial measurements were summarized as mean ± SD or median (IQR), according to data distribution, and *P* values for paired comparisons were derived using paired t-tests or Wilcoxon signed-rank tests, as appropriate. In addition, to facilitate comparison of postoperative outcomes across decompression strategies, postoperative refractive and axial changes were further compared descriptively according to decompression extent within each follow-up window. To enhance statistical stability and mitigate subgroup imbalance, surgical procedures were classified into one-wall decompression (including LWD and MWD) and two-wall decompression (BMLD). These follow-up window summaries and decompression extent comparisons were provided for descriptive and clinical interpretation only and were not incorporated into the primary longitudinal analytical framework.

#### GEE modeling

GEE models were used as the primary analytical framework to account for unequal follow-up intervals, incomplete longitudinal observations, intra-subject correlations arising from repeated measurements, and inclusion of bilateral eyes within the same patient.

For continuous outcomes, GEE models were specified using a Gaussian family and an identity link. Follow-up time was incorporated as a continuous variable, and restricted cubic splines (RCS) with three knots at the 10th, 50th, and 90th percentiles were used to capture potential nonlinear patterns. The overall effects were evaluated using joint Wald tests combining linear and nonlinear components. When the nonlinear component was not statistically significant or the spline estimation was unstable due to limited variability or substantial clustering within the variable distribution, simplified linear specifications were applied to ensure model parsimony and robustness. For nonlinear associations, critical extremum points (peaks or troughs) were identified where the first derivative of the spline function was zero [45–47].

For binary outcomes (presence versus absence of clinically significant postoperative myopic drift), logistic GEE models with a binomial family and a logit link were fitted [48, 49]. Clinically significant postoperative myopic drift was defined at each postoperative visit as a myopic change in SE (ΔSE) of ≤ − 0.50 D relative to the preoperative baseline, a threshold widely used to denote clinically meaningful refractive change and one that exceeds the expected test–retest variability of refraction [50, 51]. This outcome was analyzed as a repeated binary variable in the longitudinal GEE models.

Across all GEE models, the patient identifier was specified as the clustering variable. An autoregressive working correlation structure of order 1 [AR(1)] was adopted to account for intra-subject dependence, and robust (sandwich) variance estimators were used to ensure valid statistical inference [48, 49].

#### Covariate adjustment strategy

Covariate adjustment was prespecified based on a conceptual framework that distinguished baseline confounders from postoperative variables to minimize overadjustment and inappropriate adjustment of intermediate variables. Unless otherwise specified, all primary models were adjusted for clinically relevant confounders, including age, sex, baseline ocular parameters (AL, CCT, biomechanically corrected IOP [bIOP], and CAS), thyroid function indices (FT3, FT4, and TSH), postoperative follow-up time and orbital fat removal volume.

Covariate sets were tailored for specific models due to variations in analytical objectives. Baseline SE was excluded from longitudinal models of postoperative refractive change to prevent mathematical coupling, but was included in logistic models evaluating binary refractive outcomes [52]. Baseline exophthalmos was adjusted for in models assessing impacts of decompression strategies on postoperative ΔSE, as well as in models evaluating associations between baseline ocular biometric parameters and risks of clinically significant postoperative myopic drift. Postoperative changes in eyelid position parameters (ΔPFH and ΔMRD1), which may reflect underlying biomechanical alterations, were adjusted for in the models investigating associations between postoperative ocular biometric parameter changes and postoperative refractive change to account for the potential effects of eyelid tension. Sensitivity analyses were performed by excluding thyroid function indices from the covariate sets to assess the robustness of model estimates. Detailed covariate adjustment strategies for each analytical objective are summarized in Additional File 3.

#### Longitudinal analysis of postoperative refractive, axial, and ocular positional parameters

Longitudinal trajectories of postoperative refractive outcomes (DS, cylindrical diopter [DC], and SE), AL, and exophthalmos were analyzed using multivariable GEE models based on all available postoperative measurements. Repeated observations were modeled at the eye level and clustered within patients, and temporal patterns were characterized using RCS-based GEE models as specified above. Smoothed population-averaged trajectories with overlaid individual observations were generated to visualize longitudinal changes.

#### Dual analytical framework for evaluating overall and magnitude-adjusted effects of decompression strategies on postoperative refractive change

Beyond descriptive subgroup comparisons, longitudinal inferential analyses were additionally performed to evaluate associations between decompression strategies and postoperative refractive outcomes at both the population-averaged and temporal levels. Two complementary analytical frameworks were applied to distinguish the overall clinical effects from strategy-specific effects independent of decompression extent [53]. Analyses were structured according to exposure granularity (extent-level vs. procedure-level) and analytical objectives (overall effects vs. magnitude-adjusted effects).

The primary framework evaluated the overall effect of decompression strategy, defined as the refractive change associated with each decompression strategy as implemented in routine clinical practice. Under this framework, postoperative exophthalmos reduction (Δexophthalmos) was considered an integral component of the surgical effect, as the achieved degree of decompression inherently reflects procedural characteristics. Adjustment for Δexophthalmos was therefore not performed to avoid overadjustment of treatment-mediated pathways [54].

The secondary framework aimed to isolate strategy-specific effects independent of decompression extent. Under this framework, the achieved Δexophthalmos was included as a covariate to account for variability in decompression extent. Δexophthalmos was used as a clinically relevant indicator of decompression extent, as reduction in proptosis represents a primary and quantifiable outcome of orbital decompression surgery [55]. This adjustment enabled differentiation between the effects attributable to decompression extent and those potentially related to intrinsic procedural characteristics. Results of these complementary analytical approaches were reported separately to facilitate mechanistic interpretation.

Postoperative refractive changes were compared across decompression strategies using three complementary GEE modeling strategies [48, 49]. To compare differences in the magnitude and temporal pattern of postoperative ΔSE (continuous outcome) across decompression extent, two sets of Gaussian GEE models with identity links were fitted. First, simplified main-effects GEE models were developed to estimate the independent, population-averaged effect of decompression strategy on postoperative ΔSE across the entire follow-up period. Second, interaction GEE models incorporating a decompression strategy × follow-up time interaction term were fitted to evaluate the time-dependent differences in postoperative refractive changes across decompression strategies. Statistically significant interactions were interpreted as evidence of differential rates of postoperative refractive changes. Extent-specific temporal slopes were derived by combining the interaction term with the main time coefficient. To further assess the clinical relevance of these refractive changes, the risk of clinically significant postoperative myopic drift (binary outcome) was evaluated using multivariate logistic GEE models with binomial family and logit links.

Postoperative follow-up time was modeled as a linear continuous variable without spline terms in all models to support parsimonious inference on both average effects and temporal rates of change. To enhance statistical stability and mitigate subgroup imbalance, primary analyses were performed at the extent level (one-wall vs. two-wall decompression), with the one-wall group serving as the reference category. Exploratory procedure-level analyses (stratified by BMLD, LWD, and MWD) were additionally conducted using the main-effects models and interaction models described above under two complementary analytical frameworks (with vs. without adjustment for Δexophthalmos), to further distinguish overall clinical effects from procedure-specific effects at the individual procedure level (Supplementary Methods S1 in Additional File 4). All models were adjusted according to the prespecified covariate adjustment framework described above.

#### Investigation of associations between postoperative ocular biometric changes and postoperative refractive change

To evaluate associations between postoperative changes in ocular biometric parameters (Δparameters) and continuous postoperative ΔSE while enabling comparison across heterogeneous measurement scales, multivariable GEE models were fitted with each Δparameter standardized prior to analysis and analyzed in a separate model to reduce multicollinearity [46, 48, 49]. GEE models were specified using a Gaussian family and an identity link. RCS functions were used to assess potential nonlinear patterns, and joint Wald tests were applied to evaluate the overall effects. Absolute adjusted RCS regression coefficients (|adjusted RCS β|) were used to quantify the relative association strength and establish a hierarchical ranking of Δparameters into five tiers: dominant (> 1.0), large (0.5–1.0), medium (0.2–0.5), small (0.1–0.2), and very small (< 0.1) [56].

Moreover, non-standardized RCS-based GEE models were fitted to further explore the exposure–response relationships between Δparameters and postoperative refractive changes on the original measurement scales. Associations were visualized as smoothed exposure–response curves and classified as U-shaped, inverted U-shaped, or monotonic. Extremum points were identified where the first derivative was zero.

Detailed model specifications and simplification criteria were consistent with "GEE modeling" section and Supplementary Methods S2 in Additional File 4. All models were adjusted according to the pre-specified covariate framework described in "Covariate adjustment strategy" section. Postoperative changes in eyelid position parameters (ΔPFH and ΔMRD1) were included to account for potential effects of eyelid tension. Sensitivity analyses excluding thyroid function indices were performed within the standardized GEE framework (Supplementary Methods S3 in Additional File 4).

#### Exploration of the relationship between globe displacement and optical axial change

Nonstandardized RCS-based GEE models with a Gaussian distribution and identity link were applied to explore the association between Δexophthalmos (independent variable) and ΔAL (dependent variable). Potential nonlinear associations were assessed using RCS with three knots at the 10th, 50th, and 90th percentiles, and the overall effects were evaluated using joint Wald tests. This model was adjusted for clinically relevant confounders, including age, sex, baseline ocular parameters (AL, CCT, bIOP, CAS, and exophthalmos), thyroid function indices, postoperative follow-up time and orbital fat removal volume. Results were visualized using smoothed exposure–response curves.

#### Identification of baseline ocular biometric predictors of postoperative refractive change

Two complementary analytical frameworks were applied to identify the baseline ocular biometric predictors of postoperative refractive change.

First, the associations between individual baseline ocular biometric parameters and the risk of clinically significant postoperative myopic drift were assessed using logistic GEE models, with each parameter analyzed in a separate model to reduce multicollinearity. Continuous variables were standardized, and odds ratios (ORs) represent the change in risk per one-SD increase for each parameter [57]. Multicollinearity was assessed using variance inflation factors (VIFs) derived from equivalent linear regression models, with VIF < 5 indicating negligible collinearity [58].

Second, non-standardized RCS-based GEE models were fitted to further explore exposure–response relationships between baseline ocular biometric parameters and continuous postoperative ΔSE. Each baseline parameter was analyzed using a separate model to reduce multicollinearity.

Detailed model specifications and simplification criteria were consistent with "GEE modeling" section and Supplementary Methods S4 in Additional File 4. All models were adjusted according to the pre-specified covariate framework described in "Covariate adjustment strategy" section. Sensitivity analyses excluding thyroid function indices were conducted within the logistic GEE framework (Supplementary Methods S5 in Additional File 4).

#### Statistical analysis and sample size adequacy assessment

Given the retrospective design of this study, no formal a priori sample-size calculations were performed. The study size was determined based on the availability of eligible patients with complete longitudinal clinical data during the study period. The analytical dataset comprised repeated measurements obtained from eyes clustered within patients, and all statistical inference was conducted within a GEE framework accounting for intra-patient correlations. To provide contextual interpretation, post hoc sample size adequacy assessments for selected paired and longitudinal analyses were explored using G*Power 3.1 [59] and are presented in Supplementary Analysis S6 in Additional File 4. These calculations were intended solely as heuristic benchmarks under simplified assumptions, and did not represent formal power analyses for the GEE-based models employed in this study.

All statistical analyses and data visualization were conducted using R software (version 4.4.2) [60]. GEE models were fitted using the geepack package [61]. RCS functions and related diagnostics were implemented using the rms package [46]. Multicollinearity diagnostics were performed using the car package [62]. Graphical visualization was generated using ggplot2 [63]. All hypothesis tests were two-tailed, and statistical significance was set at* P* < 0.05.

## Results

### Baseline characteristics and follow-up distribution

Baseline characteristics of 57 eyes from 46 patients with moderate-to-severe TED are summarized in Table 1, and a complete profile is provided in Additional File 5. The cohort had a mean age of 38.8 ± 11.1 years, and 41.3% of patients were male. Ocular laterality was evenly distributed between right (50.9%) and left (49.1%) eyes. Regarding TED-related clinical features, disease activity was generally inactive, with a median CAS of 1 (IQR: 1 to 2), and only 8.8% of eyes (n = 5) had CAS ≥ 3. The mean exophthalmos was 23.0 ± 3.0 mm. The median PFH and MRD1 were 12 mm (IQR: 11 to 14 mm) and 5 mm (IQR: 4 to 7 mm), respectively. For surgical interventions, most eyes underwent BMLD (66.7%), followed by MWD (22.8%) and LWD (10.5%). The median orbital fat removal volume was 1.0 mL (IQR: 1.0 to 1.5 mL). The preoperative refractive status indicated mild myopia, with a DS of − 1.18 ± 1.76 D, DC of − 0.75 D (IQR: − 1.50 to − 0.25 D), and SE of − 1.57 ± 1.91 D. The median AL was 23.75 mm (IQR: 23.25 to 24.70 mm).

**Table 1:** Baseline demographic, clinical, surgery-related, and refractive parameters of the overall cohort

Baseline variables	Overall cohort (46 patients, 57 eyes)
Demographic variables (patient level, n = 46)	
Age (years)	38.8 ± 11.1
Sex, n (%)	
Male	19 (41.3%)
Female	27 (58.7%)
Clinical variables (eye level, n = 57)	
Ocular laterality, n (%)	
Right	29 (50.9%)
Left	28 (49.1%)
CAS	1 (1 to 2)
CAS ≥ 3, n (%)	5 (8.8%)
Exophthalmos (mm)	23.0 ± 3.0
PFH (mm)	12 (11 to 14)
MRD1 (mm)	5 (4 to 7)
Surgery-related variables (eye level, n = 57)	
Orbital fat removal volume (mL)	1.0 (1.0 to 1.5)
Surgical approach, n (%)	
LWD	6 (10.5%)
MWD	13 (22.8%)
BMLD	38 (66.7%)
Refractive and axial parameters (eye level, n = 57)	
DS (D)	− 1.18 ± 1.76
DC (D)	− 0.75 (− 1.50 to − 0.25)
SE (D)	− 1.57 ± 1.91
AL (mm)	23.75 (23.25 to 24.70)

A total of 46 patients contributed 57 eyes with unilateral and bilateral cases. Demographic variables were defined at the patient level, whereas clinical, surgical, and refractive variables were defined at the eye level. Continuous variables were presented as mean ± SD or median (IQR), depending on their distribution as assessed by the Shapiro–Wilk test, while categorical variables were summarized as counts and percentages

*AL* = axial length; *BMLD* = balanced mediolateral wall decompression; *CAS* = Clinical Activity Score; *DC* = cylindrical diopter; *DS* = spherical diopter; *IQR* = interquartile range; *LWD* = lateral wall decompression; *MRD1* = margin reflex distance 1; *MWD* = medial wall decompression; *PFH* = palpebral fissure height; *SD* = standard deviation; *SE* = spherical equivalent

**Fig. 2:**
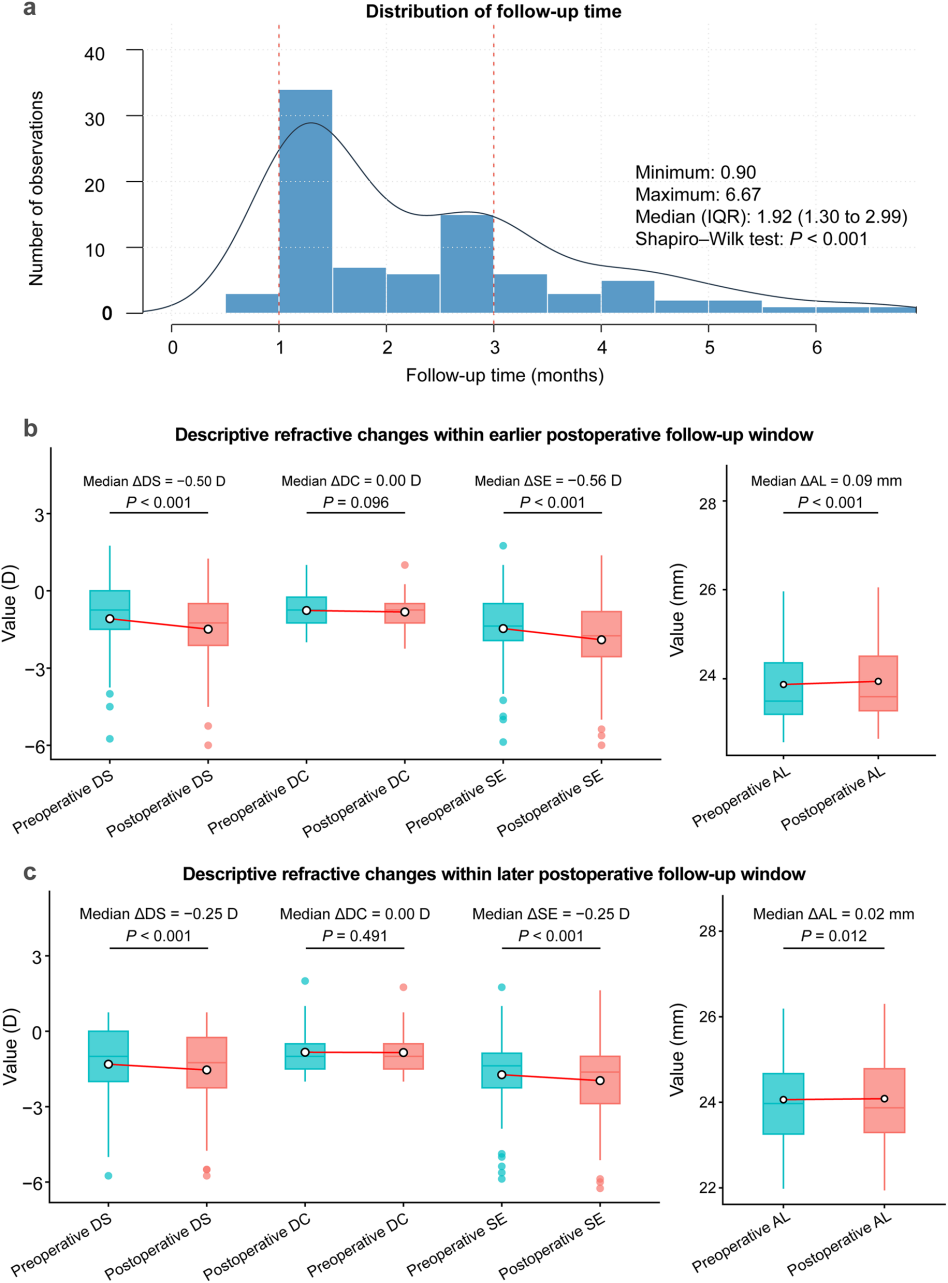
Distribution of postoperative follow-up time and descriptive visualization of refractive and axial changes across routine postoperative follow-up windows. **a** Histogram with an overlaid kernel density curve showing the distribution of postoperative follow-up time (months). Vertical dashed lines indicate routine clinical follow-up time points at approximately 1 and 3 months after surgery. Each bar represents a 0.5-month interval. The density curve was scaled to align with histogram counts. **b–c** Boxplots illustrating preoperative and postoperative refractive and axial measurements within the earlier (**b**) and later (**c**) postoperative follow-up windows. Boxplots display the median (horizontal line within the box), IQR (upper and lower box limits), and whiskers extending to 1.5 × IQR. Individual mean values are indicated by black-edged circles, and preoperative and postoperative means are connected by red lines to illustrate directional changes. *P* values for pairwise comparisons were derived from paired t-tests or Wilcoxon signed-rank tests, as appropriate. The magnitude of postoperative change (Δ) was quantified as the difference between postoperative and preoperative measurements and summarized as median (IQR). **b** Earlier postoperative follow-up window (< 2 months after surgery; 44 eyes from 34 patients; median follow-up 1.30 [IQR: 1.13 to 1.37] months). **c** Later postoperative follow-up window (≥ 2 months after surgery; 42 eyes from 34 patients; median follow-up 3.05 [IQR: 2.73 to 4.26] months). AL, axial length; DC, cylindrical diopter; DS, spherical diopter; IQR, interquartile range; SE, spherical equivalent

The distribution of postoperative follow-up time is shown in Fig. 2a. The median follow-up time was 1.92 (IQR: 1.30 to 2.99) months, with a range of 0.90 to 6.67 months. The distribution was right-skewed, and the Shapiro–Wilk test indicated a significant deviation from normality (*P* < 0.001). Visual inspection revealed broad clustering of observations around approximately 1- and 3-month postoperative periods, consistent with routine clinical follow-up patterns, whereas substantial inter-individual variability in follow-up timing was observed.

Corresponding to these follow-up patterns, postoperative observations were additionally summarized descriptively within the predefined earlier and later postoperative follow-up windows (Fig. 2b, c; Table 2). The earlier postoperative window included 44 eyes from 34 patients with a median follow-up of 1.30 (IQR: 1.13 to 1.37) months, whereas the later postoperative window included 42 eyes from 34 patients with a median follow-up of 3.05 (IQR: 2.73 to 4.26) months. In the earlier postoperative window, postoperative DS and SE demonstrated median changes of − 0.50 D (IQR: − 0.75 to − 0.25 D) and − 0.56 D (IQR: − 0.66 to − 0.25 D), respectively. Postoperative AL measurements demonstrated a median increase of 0.09 mm (IQR: 0.05 to 0.11 mm). In the later postoperative window, the magnitude of postoperative change was attenuated, with median ΔDS, ΔSE, and ΔAL values of − 0.25 D (IQR: − 0.25 to 0.00 D), − 0.25 D (IQR: − 0.47 to − 0.13 D), and 0.02 mm (IQR: − 0.02 to 0.06 mm), respectively. Median postoperative ΔDC remained minimal across both follow-up windows. Clinically significant postoperative myopic drift (ΔSE ≤ − 0.50 D) was observed in 63.6% of eyes during the earlier postoperative window and in 26.2% during the later postoperative window. Compared with the earlier postoperative window, these findings suggest partial recovery and a trend toward relative stabilization of postoperative refractive changes over time.

**Table 2:** Descriptive summary of postoperative refractive and axial changes across postoperative follow-up windows

Refractive and axial outcomes	Preoperative	Postoperative	Δ (Post − Pre)
Earlier postoperative window (44 eyes from 34 patients)			
DS (D)	− 0.98 ± 1.70	− 1.39 ± 1.76	− 0.50 (− 0.75 to − 0.25)
DC (D)	− 0.75 ± 0.70	− 0.81 ± 0.70	0.00 (− 0.25 to 0.06)
SE (D)	− 1.36 ± 1.83	− 1.79 ± 1.91	− 0.56 (− 0.66 to − 0.25)
AL (mm)	23.50 (23.20 to 24.36)	23.60 (23.28 to 24.50)	0.09 (0.05 to 0.11)
Later postoperative window (42 eyes from 34 patients)			
DS (D)	− 1.00 (− 2.19 to − 0.06)	− 1.25 (− 2.25 to − 0.25)	− 0.25 (− 0.25 to 0.00)
DC (D)	− 1.00 (− 1.50 to − 0.50)	− 1.00 (− 1.50 to − 0.50)	0.00 (− 0.25 to 0.19)
SE (D)	− 1.38 (− 2.34 to − 0.88)	− 1.63 (− 2.97 to − 1.00)	− 0.25 (− 0.47 to − 0.13)
AL (mm)	24.06 ± 1.06	24.09 ± 1.08	0.02 (− 0.02 to 0.06)

The earlier and later postoperative follow-up windows corresponded to < 2 and ≥ 2 months after surgery, respectively. Preoperative and postoperative refractive and axial measurements were presented as mean ± SD or median (IQR) according to data distribution within each follow-up window. Δ (Post − Pre) was calculated as postoperative minus preoperative values and summarized as median (IQR)

*AL* = axial length; *DC* = cylindrical diopter; *DS* = spherical diopter; *IQR* = interquartile range; *SD* = standard deviation; *SE* = spherical equivalent

### Orbital decompression was associated with nonlinear temporal changes in ocular refraction, AL, and globe position

**Fig. 3:**
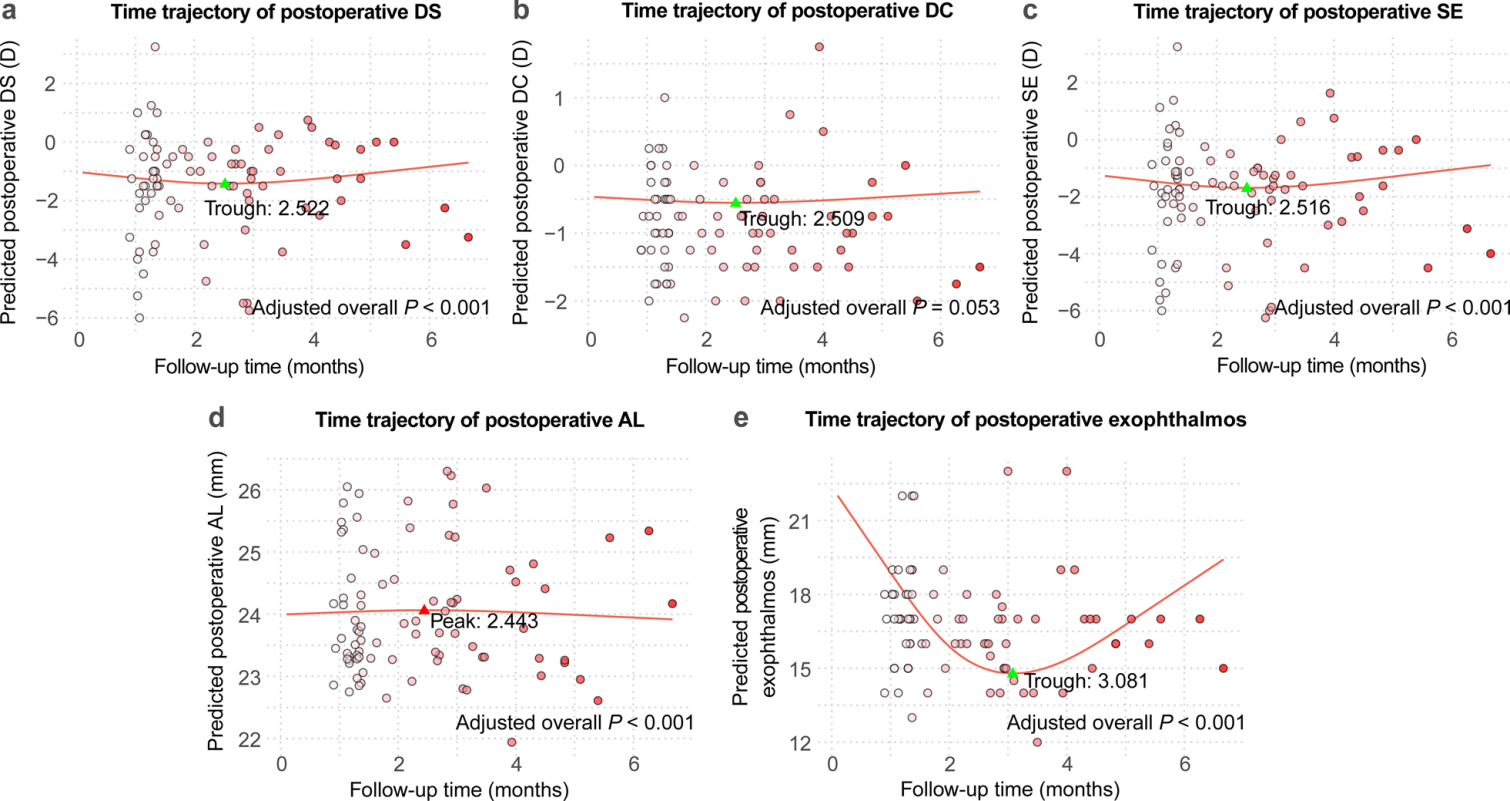
Longitudinal trajectories of postoperative refractive, axial, and ocular positional parameters following orbital decompression. Population-averaged trajectories of postoperative (**a**) DS, (**b**) DC, (**c**) SE, (**d**) AL, and (**e**) exophthalmos derived from RCS-based GEE models and visualized as smoothed curves. Individual observations were overlaid with color intensity representing follow-up time. AL, axial length; DC, cylindrical diopter; DS, spherical diopter; GEE, generalized estimating equations; RCS, restricted cubic splines; SE, spherical equivalent

Population-averaged longitudinal GEE modeling demonstrated significant nonlinear temporal trajectories for postoperative DS and SE (Fig. 3a, c; Table 3; both *P* < 0.001), with a progressive increase in myopic shift during the early postoperative period, reaching a maximum at approximately 2–3 months postoperatively, followed by partial hyperopic recovery thereafter. In contrast, postoperative DC demonstrated only mild transient changes that did not reach statistical significance (Fig. 3b; *P* = 0.053).

**Table 3:** Longitudinal trajectories of postoperative refractive, axial, and ocular positional parameters following orbital decompression

Postoperative variables	Linear component		Non-linear component		Adjusted overall *P* value	Extremum timing (months)	Temporal pattern
	Adjusted β (95% CI)	Adjusted* P* value	Adjusted RCS β	Adjusted *P* value			
DS (D)	− 0.207 (− 0.27 to − 0.14)	** < 0.001**	0.533	** < 0.001**	** < 0.001**	Trough at 2.52	Initial myopic shift followed by partial recover
DC (D)	− 0.048 (− 0.09 to − 0.01)	**0.016**	0.123	**0.041**	0.053	Trough at 2.51	Mild transient astigmatic change
SE (D)	− 0.229 (− 0.30 to − 0.16)	** < 0.001**	0.592	** < 0.001**	** < 0.001**	Trough at 2.52	Initial myopic shift followed by partial recover
AL (mm)	0.037 (0.02 to 0.05)	** < 0.001**	− 0.102	** < 0.001**	** < 0.001**	Peak at 2.44	Initial increase in measured AL followed by partial recover
Exophthalmos (mm)	− 3.477 (− 3.86 to − 3.09)	** < 0.001**	6.352	** < 0.001**	** < 0.001**	Trough at 3.08	Initial posterior globe displacement followed by partial rebound

Longitudinal changes in DS, DC, SE, AL, and exophthalmos were estimated using RCS-based GEE models. Linear and nonlinear spline components are shown separately, with adjusted overall *P* values derived from joint Wald tests. Extremum timing represents the estimated postoperative time point corresponding to the extremum (peak or trough) of the fitted trajectory. Temporal pattern descriptions are intended to facilitate clinical interpretation of the longitudinal trajectories. All estimates were adjusted for age, sex, baseline ocular parameters (AL, CCT, bIOP, and CAS), thyroid function indices, and orbital fat removal volume. Postoperative follow-up time was incorporated as the time scale and modeled using RCS. Bold values indicate statistical significance (*P* < 0.05)

*AL* = axial length; *bIOP* = biomechanically corrected intraocular pressure; *CAS* = Clinical Activity Score; *CCT* = central corneal thickness; *CI* = confidence interval; *DC* = cylindrical diopter; *DS* = spherical diopter; *GEE* = generalized estimating equations; *RCS* = restricted cubic splines; *SE* = spherical equivalent; *β* = regression coefficient

In parallel, postoperative AL measurements demonstrated a significant nonlinear temporal pattern (Fig. 3d; *P* < 0.001), with maximal postoperative increase occurring at approximately 2–3 months followed by partial reversal thereafter. Postoperative exophthalmos exhibited a similar nonlinear trajectory (Fig. 3e; *P* < 0.001), characterized by maximal posterior globe displacement during the early postoperative period followed by partial anterior rebound over time.

Collectively, these findings demonstrated that orbital decompression was associated with a predominantly spherical myopic shift that peaked at approximately 2–3 months postoperatively and partially recovered thereafter, with postoperative refractive status gradually approaching relative stabilization over time, whereas cylindrical refractive changes remained comparatively limited.

### Decompression strategies influence postoperative refractive change through both magnitude-dependent and strategy-specific effects

Descriptive comparisons across routine postoperative follow-up windows demonstrated consistent extent-dependent differences in postoperative refractive outcomes (Table 4). In both the earlier and later postoperative windows, two-wall decompression showed greater myopic shifts in DS and SE, along with larger postoperative increases in measured AL, compared with one-wall decompression. The proportion of eyes exhibiting clinically significant postoperative myopic drift was also substantially higher in the two-wall subgroup across both follow-up windows. These descriptive findings provided clinically intuitive support for the subsequent longitudinal GEE analyses.

**Table 4:** Descriptive comparison of postoperative refractive and axial changes according to decompression extent across postoperative follow-up windows

Earlier postoperative window (44 eyes from 34 patients)			
Outcome	One-wall decompression (14 eyes)	Two-wall decompression (30 eyes)	*P* value
ΔDS (D)	− 0.125 (− 0.438 to 0.250)	− 0.750 (− 0.750 to − 0.500)	** < 0.001**
ΔDC (D)	0.000 (− 0.188 to 0.250)	− 0.125 (− 0.250 to 0.000)	0.136
ΔSE (D)	0.000 (− 0.375 to 0.344)	− 0.625 (− 0.875 to − 0.531)	** < 0.001**
ΔAL (mm)	0.060 (0.022 to 0.070)	0.110 (0.090 to 0.125)	** < 0.001**
Clinically significant myopic drift, n (%)	2 (14.3)	26 (86.7)	** < 0.001**

Later postoperative window (42 eyes from 34 patients)			
Outcome	One-wall decompression (16 eyes)	Two-wall decompression (26 eyes)	*P* value
ΔDS (D)	− 0.125 (− 0.250 to 0.000)	− 0.250 (− 0.500 to − 0.250)	**0.004**
ΔDC (D)	0.000 (− 0.250 to 0.250)	0.000 (− 0.250 to 0.000)	0.273
ΔSE (D)	− 0.125 (− 0.250 to 0.125)	− 0.250 (− 0.625 to − 0.125)	**0.003**
ΔAL (mm)	0.005 (− 0.025 to 0.020)	0.050 (0.020 to 0.090)	**0.016**
Clinically significant myopic drift, n (%)	0 (0.0)	11 (42.3)	**0.003**

Earlier and later postoperative follow-up windows corresponded to < 2 months and ≥ 2 months after surgery, respectively. For consistency of presentation across groups, continuous variables were presented as median (IQR) and compared using the Mann–Whitney U test. Categorical variables were compared using Pearson’s chi-squared test (with Yates’ continuity correction) or Fisher’s exact test, as appropriate. Clinically significant myopic drift was defined as ΔSE ≤ − 0.50 D. Δ values were calculated relative to preoperative baseline

*AL =* axial length; *DC* = cylindrical diopter; *DS* = spherical diopter; *IQR* = interquartile range; *SE* = spherical equivalent

#### Overall associations of decompression strategies with postoperative refractive change

In the overall-effects framework reflectingroutine clinical practice, decompression strategy demonstrated significant associations with postoperative refractive outcomes across magnitude, temporal progression, and clinical risk, extending the descriptive patterns observed in Table 4.

In the population-averaged main-effects model (Table 5a), two-wall decompression was associated with a significantly greater myopic shift compared with one-wall decompression (β = − 0.279 D, *P* < 0.001). Follow-up time was also independently associated with progressive myopic change (β = − 0.051 D/month, *P* < 0.001), indicating cumulative myopic shift over time.

**Table 5:** Extent-level effects of decompression strategies on postoperative refractive change estimated under complementary frameworks

a Main-effects GEE model								
Variable	Overall effects				Magnitude-adjusted effects			
	β (D)	StdErr	*P* value	95% CI	β (D)	StdErr	*P* value	95% CI
Two-wall vs. One-wall	− 0.2788	0.046	** < 0.001**	− 0.368 to − 0.189	− 0.2772	0.046	** < 0.001**	− 0.367 to − 0.188
Follow-up time (months)	− 0.0514	0.012	** < 0.001**	− 0.074 to − 0.029	− 0.0004	0.019	0.983	− 0.037 to 0.036

b Interaction GEE model								
Variable	Overall effects				Magnitude-adjusted effects			
	β (D/month)	StdErr	*P* value	95% CI	β (D/month)	StdErr	*P* value	95% CI
Two-wall × Time vs. One-wall × Time	− 0.1773	0.054	** < 0.001**	− 0.282 to − 0.072	− 0.2169	0.047	** < 0.001**	− 0.309 to − 0.125

c Extent-specific temporal slopes		
Decompression extent	Overall effects	Magnitude-adjusted effects
	Slope (D/month)	Slope (D/month)
One-wall	− 0.0514	− 0.0004
Two-wall	− 0.2287	− 0.2173

d Logistic GEE model								
Variable	Overall effects				Magnitude-adjusted effects			
	β (log odds)	StdErr	*P* value	OR (95% CI)	β (log odds)	StdErr	*P* value	OR (95% CI)
Two-wall vs. One-wall	2.9029	0.679	** < 0.001**	18.236 (4.820 to 68.936)	2.4042	0.341	** < 0.001**	11.070 (5.677 to 21.583)

Decompression strategies were categorized as one- or two-wall decompression, with results presented under overall and magnitude-adjusted analytical frameworks. (a) Main-effects GEE models estimating the independent population-averaged effects of decompression extent and follow-up time on postoperative ΔSE. (b) Interaction GEE models evaluating differential temporal trajectories of postoperative ΔSE through inclusion of the decompression extent × follow-up time interaction term. (c) Extent-specific temporal slopes represent the estimated monthly rates of refractive change within each subgroup and were derived by summing the main-effects time coefficient and interaction term. (d) Logistic GEE models evaluating the association between decompression extent and the risk of clinically significant postoperative myopic drift. The one-wall decompression group served as the reference category for all models. All models were adjusted for age, sex, baseline ocular parameters (AL, CCT, bIOP, CAS, and exophthalmos), thyroid function indices, and orbital fat removal volume. Postoperative follow-up time was incorporated as an explicitly modeled temporal predictor. Δexophthalmos was additionally adjusted in the magnitude-adjusted analytical framework but not in the overall analytical framework. β values and slope estimates are presented to four decimal places because of the low magnitude of certain values (|value| < 0.0005), in order to preserve numerical precision. Bold values indicate statistical significance (*P* < 0.05)

*AL* = axial length; *bIOP* = biomechanically corrected intraocular pressure; *CAS* = Clinical Activity Score; *CCT* = central corneal thickness; *CI* = confidence interval; *GEE* = generalized estimating equations; *OR* = odds ratio; *SE* = spherical equivalent; *StdErr* = standard error; *β* = regression coefficient

The interaction GEE model (Table 5b) further demonstrated a significant interaction between decompression strategy and follow-up time, indicating differential temporal trajectories across decompression strategies. Two-wall decompression was associated with a significantly steeper rate of myopic progression over time than one-wall decompression (β = − 0.177 D/month, *P* < 0.001). Extent-specific slope analysis (Table 5c) further demonstrated that the one-wall subgroup showed a negligible monthly refractive change (− 0.051 D/month), whereas the two-wall subgroup exhibited a markedly steeper myopic progression rate (− 0.229 D/month). These findings were consistent with the greater decompression extent achieved by two-wall decompression.

The logistic GEE model (Table 5d) indicated that two-wall decompression was associated with significantly higher risks of clinically significant postoperative myopic drift relative to one-wall decompression (odds ratio [OR] = 18.236, 95% CI: 4.820 to 68.936, *P* < 0.001). Collectively, these findings indicate that decompression extent influences both the magnitude and temporal dynamics of postoperative refractive change.

#### Magnitude-adjusted associations independent of decompression extent

After adjustment for Δexophthalmos, the association between decompression strategy and postoperative refractive change remained evident, although temporal patterns were altered.

The adjusted main-effects GEE model (Table 5a) revealed that two-wall decompression continued to demonstrate a significantly greater myopic shift than one-wall decompression (β = − 0.277 D, *P* < 0.001), with minimal effect size change from the unadjusted model (β = − 0.279 D). In contrast, the previously observed time-dependent progression was no longer evident (β = − 0.0004 D/month, *P* = 0.983), suggesting that the time-dependent refractive change may be largely related to decompression extent.

The adjusted interaction model (Table 5b) demonstrated that the significant interaction between decompression strategy and follow-up time persisted after adjustment for Δexophthalmos. Two-wall decompression continued to demonstrate significantly steeper refractive progression than one-wall decompression (β = − 0.217 D/month, *P* < 0.001), with a slightly larger interaction effect size than in the unadjusted model (β = − 0.177 D/month). The slope analysis further clarified this pattern (Table 5c). The one-wall subgroup exhibited near-zero monthly refractive change after adjustment (slope = − 0.0004 D/month), whereas the two-wall subgroup maintained pronounced progressive myopic shift (slope = − 0.217 D/month).

In the adjusted logistic model (Table 5d), two-wall decompression remained associated with higher risks of clinically significant myopic drift (OR = 11.070, 95% CI: 5.677 to 21.583, *P* < 0.001) compared with one-wall decompression, although the association was stronger in the unadjusted model (OR = 18.236).

#### Procedure-level exploratory analyses

Exploratory comparisons of individual decompression procedures revealed patterns consistent with the primary extent-level findings (Supplementary Results S1 in Additional File 4). BMLD consistently exhibited the largest magnitude and fastest rate of myopic drift across all models. MWD showed modest myopic change in unadjusted analyses, which shifted toward slight hyperopic change after adjustment for Δexophthalmos. LWD demonstrated intermediate patterns across analyses. Given the subgroup size imbalance, these procedure-level findings should be interpreted as associative and hypothesis generating.

Collectively, these results indicate that postoperative refractive change is jointly shaped by decompression extent and the intrinsic properties of surgical procedures.

### Hierarchical associations identify optical axial change as the dominant correlate of postoperative refractive change

**Fig. 4:**
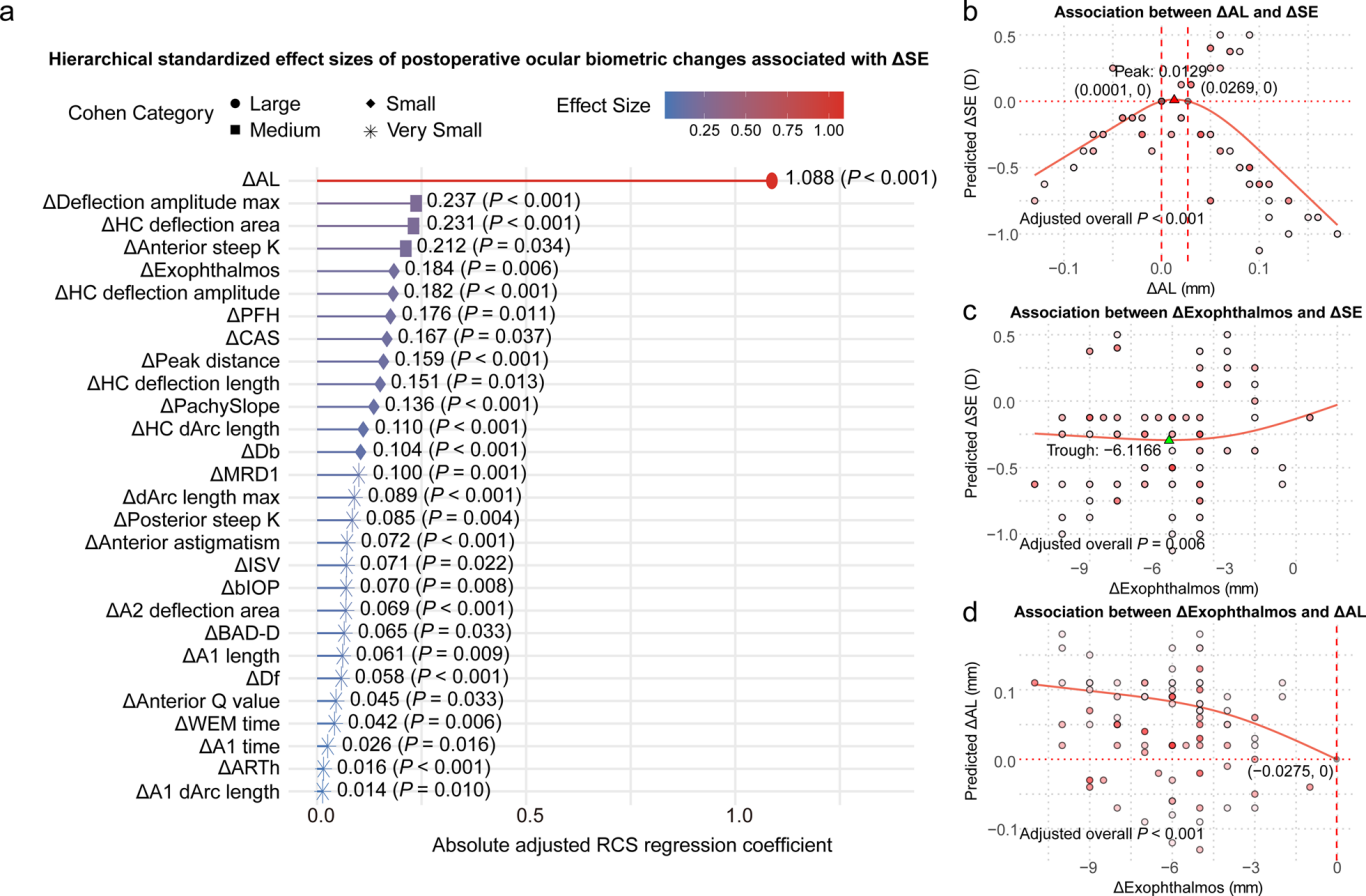
Hierarchical and exposure–response relationships between postoperative ocular biometric changes and postoperative refractive changes.** a** Hierarchical ranking of postoperative ocular biometric changes based on absolute standardized RCS regression coefficients derived from multivariable GEE models. The parameters were color-coded according to predefined effect size tiers. **b–c** Smooth curves illustrating nonlinear associations between key ocular biometric changes and continuous postoperative ΔSE using non-standardized RCS-based GEE models. **b** ΔAL showed an inverted U-shaped association with ΔSE, with the estimated peak of ΔSE indicated by a red triangle. **c** Δexophthalmos showed a U-shaped association with ΔSE, with the estimated trough of ΔSE indicated by a green triangle. Models in (**a**–**c**) were adjusted for age, sex, baseline ocular parameters (AL, CCT, bIOP, CAS), thyroid function indices, postoperative follow-up time, orbital fat removal volume, ΔPFH, and ΔMRD1. **d** Smooth curves illustrating nonlinear associations between Δexophthalmos and ΔAL, with solid lines representing adjusted fitted relationships. The model was adjusted for age, sex, baseline ocular parameters (AL, CCT, bIOP, CAS, and exophthalmos), thyroid function indices, postoperative follow-up time, and orbital fat removal volume. AL, axial length; ARTh, Ambrósio’s relational thickness (horizontal); BAD-D, Belin/Ambrósio Display D index; bIOP, biomechanically corrected intraocular pressure; CAS, Clinical Activity Score; CCT, central corneal thickness; dArc, deformation arc; Db, back elevation deviation; Df, front elevation deviation; GEE, generalized estimating equations; HC, highest concavity; ISV, index of surface variance; K, keratometry; MRD1, margin reflex distance 1; PFH, palpebral fissure height; RCS, restricted cubic splines; WEM time, maximum whole eye movement time

Standardized spline-based analyses identified a hierarchical pattern in the association between postoperative changes in ocular biometric parameters and continuous postoperative ΔSE (Fig. 4a). Complete results for all examined parameters are provided in Additional File 6.

Postoperative ΔAL showed the strongest association with ΔSE, exhibiting the largest standardized RCS effect among all evaluated parameters (|adjusted RCS β|= 1.088, *P* < 0.001), substantially exceeding the effect sizes observed for the other variables.

A limited number of parameters demonstrated medium-strength associations with ΔSE, including Δdeflection amplitude max (|adjusted RCS β|= 0.237, *P* < 0.001), Δhighest concavity (HC) deflection area (|adjusted RCS β|= 0.231, *P* < 0.001), and Δanterior steep K (|adjusted RCS β|= 0.212, *P* = 0.034), indicating meaningful, though secondary involvement of corneal biomechanical responses and anterior corneal shape changes in relation to postoperative refractive change.

Several additional parameters showed small but statistically significant associations, including Δexophthalmos, ΔHC deflection amplitude, ΔPFH, ΔCAS, Δpeak distance, ΔHC deflection length, Δpachymetry slope (PachySlope), ΔHC deformation arc (dArc) length, and Δback elevation deviation (Db) (all *P* < 0.05), spanning orbital, corneal biomechanical, and corneal tomographic parameters. A broader group of parameters demonstrated very small standardized effects despite statistical significance, including ΔMRD1, Δmaximum deformation arc length (dArc length max), Δposterior steep K, Δanterior astigmatism, Δindex of surface variance (ISV), ΔbIOP, Δsecond applanation (A2) deflection area, ΔBelin/Ambrósio Display D index (BAD-D), Δfirst applanation (A1) length, Δfront elevation deviation (Df), Δanterior Q value, Δmaximum whole eye movement (WEM) time, ΔA1 time, ΔAmbrósio’s relational thickness (ARTh), and ΔA1 dArc length (all *P* < 0.05). Among these, Δexophthalmos, ΔCAS, and ΔbIOP, although not components of the optical system and with limited direct involvement in the refractive pathway, showed significant associations with ΔSE. Of particular interest, Δexophthalmos represents an orbital structural parameter reflecting globe repositioning. The mechanism underlying this indirect contribution to postoperative refractive changes warrants further investigation.

For posterior corneal parameters with highly concentrated distributions, the spline-based GEE models could not be reliably estimated because of insufficient variability and clustering in the data. In line with our analytical protocol, these parameters were modeled using continuous linear terms to ensure stable and robust estimation. Linear GEE models revealed a significant association between Δposterior flat K and ΔSE (*P* = 0.010), whereas no significant associations were observed for Δposterior mean K or Δposterior astigmatism (both *P* > 0.05). Consequently, these parameters were excluded from the spline-based hierarchical ranking.

Nonstandardized RCS-based GEE models further characterized the nonlinear exposure–response patterns of these associations (Supplementary Results S2 in Additional File 4). Notably, ΔAL exhibited a significant nonlinear association with ΔSE (Fig. 4b; *P* < 0.001), characterized by an inverted U-shaped pattern in which intermediate axial elongation corresponded to minimal myopic shift, whereas both smaller and larger axial changes were associated with greater myopic drift. Δexophthalmos likewise demonstrated a significant nonlinear association with ΔSE (Fig. 4c; *P* = 0.006), which exhibited a U-shaped pattern with moderate reductions associated with maximal myopic drift and both minimal and extensive reductions associated with reduced drift or hyperopic shift. This pattern is consistent with a magnitude-dependent association between orbital decompression and refractive change and aligns with the pathway identified in the procedure-level analyses. Complete associations are presented in Additional File 7 and significant associations are depicted in Additional File 8.

Sensitivity analyses excluding thyroid function indices based on the standardized GEE framework yielded largely consistent hierarchical patterns (Supplementary Results S3 in Additional File 4), supporting the robustness of these hierarchical associations. Complete effect estimates and spline patterns for all parameters are provided in Additional File 9.

Taken together, these findings demonstrate a hierarchical structure of associations with postoperative ΔSE, with ΔAL showing the strongest association, alongside contributions from ocular positional, eyelid-related, corneal biomechanical, and corneal tomographic changes.

### Globe displacement is associated with optical axial change in a monotonic nonlinear manner

Non-standardized RCS-based GEE models revealed significant associations between Δexophthalmos and ΔAL (Fig. 4d; *P* < 0.001), exhibiting a monotonic downward pattern. Greater reductions in exophthalmos were associated with larger increases in optically measured AL, suggesting potential coupling between posterior globe displacement and optical axial elongation.

### Baseline ocular biometric profiles demonstrated heterogeneous associations with postoperative refractive change

**Fig. 5:**
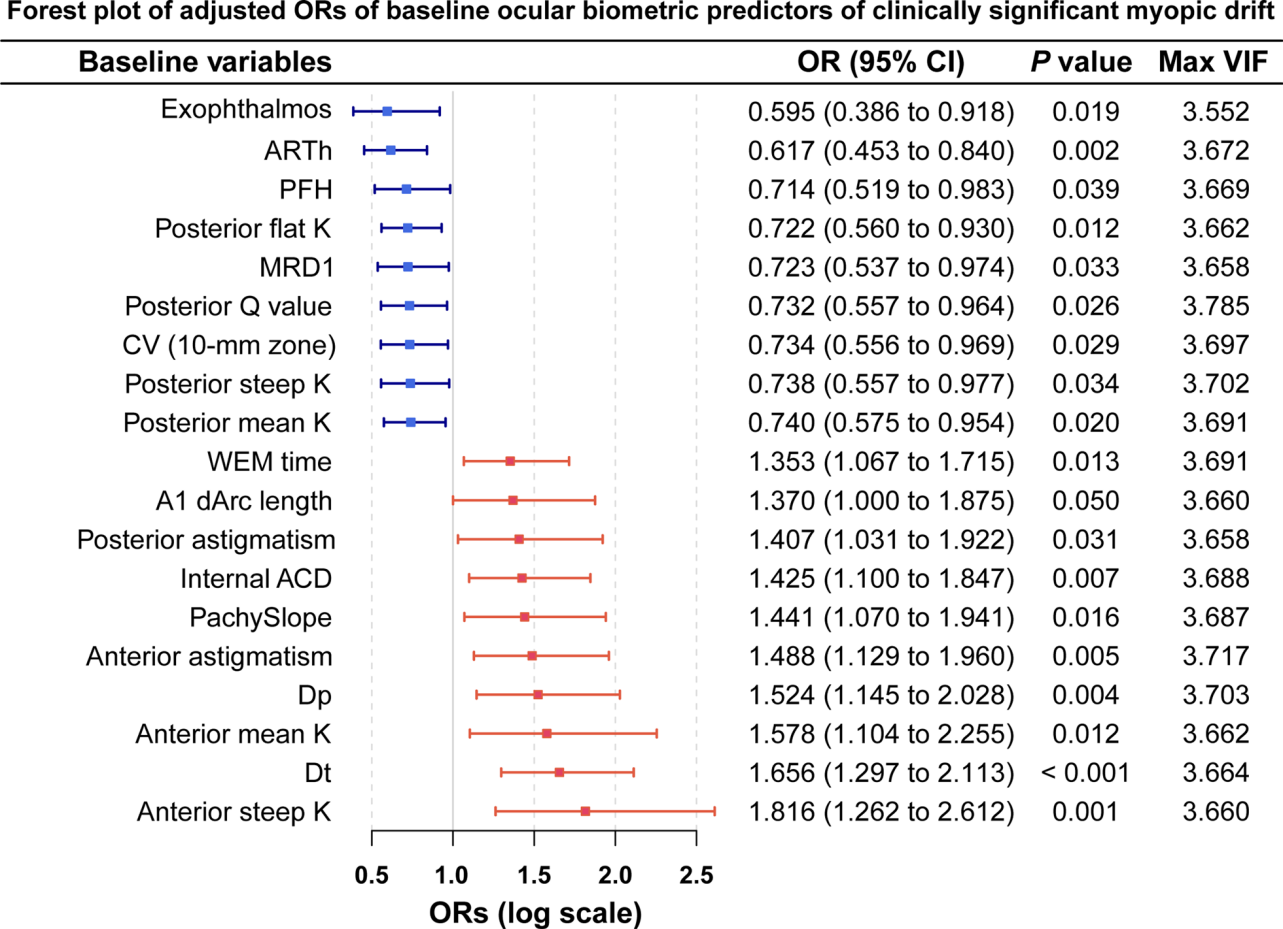
Baseline ocular biometric predictors of clinically significant postoperative myopic drift. Forest plot of adjusted ORs with 95% CI derived from multivariable logistic GEE models. The models were adjusted for age, sex, baseline ocular parameters (AL, CCT, bIOP, CAS, and exophthalmos), thyroid function indices, postoperative follow-up time, and orbital fat removal volume. ORs represent the change in the odds of clinically significant myopic drift per standard deviation increase in the corresponding baseline parameter. Each row represents a baseline parameter analyzed in an individual model. Points indicate adjusted ORs, and horizontal bars denote 95% CI.* P* values correspond to the Wald tests for the parameter of interest within each model. AL, axial length; ARTh, Ambrósio’s relational thickness (horizontal); bIOP, biomechanically corrected intraocular pressure; CAS, Clinical Activity Score; CCT, central corneal thickness; CI, confidence interval; CV, corneal volume; dArc, deformation arc; Dp, pachymetric progression index deviation; Dt, thinnest point deviation; GEE, generalized estimating equations; Internal ACD, internal anterior chamber depth; K, keratometry; Max VIF, maximum variance inflation factor; MRD1, margin reflex distance 1; OR, odds ratio; PFH, palpebral fissure height; SD, standard deviation; WEM time, maximum whole eye movement time

Logistic GEE models identified heterogeneous associations between baseline ocular biometric parameters and the risk of clinically significant postoperative myopic drift (Fig. 5). Complete results for all examined parameters are provided in Additional File 10.

Several baseline parameters were associated with a lower risk of clinically significant myopic drift. Orbital- and eyelid-related parameters, including larger baseline exophthalmos (OR = 0.595, *P* = 0.019), MRD1 (OR = 0.723, *P* = 0.033), and PFH (OR = 0.714, *P* = 0.039), were independently associated with reduced risk. Among the corneal biomechanical parameters, a more gradual corneal thickness profile (higher ARTh: OR = 0.617, *P* = 0.002) was also associated with diminished risk. Corneal morphological parameters further conferred a protective effect, including a flatter posterior corneal surface (higher [i.e., less negative] posterior flat K: OR = 0.722, *P* = 0.012; higher posterior steep K: OR = 0.738, *P* = 0.034; higher posterior mean K: OR = 0.740, *P* = 0.020), a less prolate posterior corneal shape (higher posterior Q value: OR = 0.732, *P* = 0.026), and a larger corneal volume within the 10-mm zone (larger CV: OR = 0.734, *P* = 0.029).

Conversely, several baseline parameters were associated with a higher risk of clinically significant myopic drift. Specifically, corneal biomechanical parameters, including longer WEM time (OR = 1.353, *P* = 0.013), longer A1 dArc length (OR = 1.370, *P* = 0.050), and higher PachySlope (OR = 1.441, *P* = 0.016), were independently associated with an increased risk. Corneal morphological variables, including a steeper anterior corneal surface (higher anterior steep K: OR = 1.816, *P* = 0.001; higher anterior mean K: OR = 1.578, *P* = 0.012), greater anterior corneal astigmatism (OR = 1.488, *P* = 0.005), and greater posterior corneal astigmatism (OR = 1.407, *P* = 0.031), were also correlated with an elevated risk. Additionally, a greater deviation in corneal thickness distribution (higher thinnest point deviation [Dt]: OR = 1.656, *P* < 0.001; higher pachymetric progression index deviation [Dp]: OR = 1.524, *P* = 0.004) and greater internal anterior chamber depth (internal anterior chamber depth [ACD]: OR = 1.425, *P* = 0.007) conferred a greater risk of clinically significant myopic drift. Multicollinearity diagnostics indicated no meaningful collinearity among the predictors (all VIFs were < 5), supporting the stability of these estimates.

Non-standardized RCS-based GEE models further characterized the nonlinear patterns of the associations between baseline ocular biometric parameters and continuous postoperative ΔSE (Supplementary Results S4 in Additional File 4). Complete associations are presented in Additional File 11 and significant associations are depicted in Additional File 12. Sensitivity analyses excluding thyroid function indices based on the logistic GEE framework yielded highly consistent results (Supplementary Results S5 in Additional File 4), supporting the robustness of the primary analyses. Complete results for all examined baseline parameters are provided in Additional File 13.

## Discussion

Orbital decompression remains the cornerstone intervention for moderate-to-severe TED [1, 3, 4], but its longitudinal refractive consequences have not been fully characterized. In this study, we performed a time-resolved analysis demonstrating that orbital decompression is associated with a biphasic refractive pattern that predominantly affects the spherical component, with the greatest myopic shift occurring at approximately 2–3 months postoperatively followed by partial hyperopic recovery. Greater decompression extent was consistently associated with larger refractive changes, faster temporal progression, and higher risks of clinically significant postoperative myopic drift. Standardized GEE analyses further identified ΔAL as the strongest statistical correlate of postoperative refractive changes, alongside contributions from ocular positional, eyelid-related, corneal biomechanical, and corneal tomographic changes.

Because postoperative observations clustered broadly around routine clinical visits at approximately 1 and 3 months after surgery, descriptive subgroup summaries were additionally provided within earlier and later postoperative follow-up windows to facilitate clinically intuitive interpretation. These descriptive analyses demonstrated that postoperative refractive changes were more pronounced during the earlier postoperative period and became attenuated during later follow-up.

Spline-based longitudinal modeling further clarified the timeline of refractive stabilization after surgery. Postoperative DS and SE reached their greatest myopic shifts at approximately 2–3 months after surgery, followed by gradual partial hyperopic recovery thereafter. This temporal pattern may account for previously reported variability in refractive outcomes after orbital decompression, as early postoperative measurements likely captured initial myopic shifts [7, 8], whereas later assessments may reflect a more stabilized or partially recovered refractive state [9, 10]. Moreover, the relative stability of the cylindrical components suggests that astigmatic changes are less sensitive to global biomechanical alterations. This observation is consistent with previous studies reporting the persistence of pre-existing with-the-rule astigmatism after surgery [11], suggesting limited influence of orbital manipulation on corneal shape and supporting the predominance of spherical refractive change following decompression.

Methodologically, this study extends prior work by adopting a longitudinal design with repeated postoperative measurements and applying GEE models combined with RCS. This approach accounts for intra-subject correlations and allows flexible modeling of nonlinear temporal trends [46, 48, 49], enabling dynamic characterization of postoperative refractive and axial changes compared with cross-sectional or short-term analyses. Clinically, these findings provide practical guidance regarding the timing of postoperative refractive stabilization. Definitive refractive correction, including spectacle prescription and intraocular lens power calculation, may be better deferred until at least approximately 3 months postoperatively, particularly after more extensive decompression [15].

Beyond temporal dynamics, decompression strategy was significantly associated with postoperative refractive changes through both magnitude-related and strategy-specific pathways. Under the overall analytical framework reflecting routine clinical practice, two-wall decompression demonstrated greater postoperative myopic shifts, faster temporal progression, and higher risks of clinically significant postoperative myopic drift than one-wall decompression. Under the magnitude-adjusted analytical framework, adjustment for Δexophthalmos largely eliminated the independent temporal effect observed in the main-effects model, suggesting that the apparent temporal progression of postoperative refractive change was predominantly related to decompression extent. The attenuated association between decompression strategy and clinically significant myopic drift in logistic regression models further suggested that decompression extent substantially influenced clinical refractive outcomes. However, significant interaction effects and differential temporal slopes between decompression strategies persisted after adjustment, indicating unique time-varying dynamics inherent to each strategy that are independent of decompression extent. Consistent patterns identified in exploratory comparisons of individual decompression procedures further supported this dual influence. Collectively, these results indicate that postoperative refractive changes are jointly governed by decompression extent and strategy-specific biomechanical effects.

Mechanistically, multi-wall decompression induces greater orbital volume expansion, leading to more pronounced globe displacement [12], redistribution of extraocular muscle forces [13], and altered periocular pressure gradients [64, 65]. These structural modifications remodel the overall ocular mechanical environment and establish a structural basis for postoperative refractive alterations.

Among postoperative ocular biometric parameters, ΔAL emerged as the dominant correlate of refractive change. From an optical perspective, any process that increases the effective distance between the corneal refracting surface and retinal photoreceptor plane—whether due to posterior globe displacement, whole-globe translation, or changes in globe orientation—will be registered by optical biometry as an increase in AL [29]. Accordingly, ΔAL should not be interpreted as a direct measure of true anatomical elongation in the context of orbital decompression. Rather, it may represent an integrated optical surrogate of ocular geometric remodeling, potentially reflecting the combined effects of posterior globe deformation, whole-globe translational displacement within the orbit, and rotational realignment of the visual axis relative to the corneal apex [12, 43, 66]. The nonlinear relationship between ΔAL and ΔSE likely reflects underlying multi-factor biomechanical–optical coupling, rather than a departure from classical optical principles.

This interpretation is further supported by the changing patterns of Δexophthalmos. As the most direct clinical indicator of anteroposterior globe translation [55], Δexophthalmos was significantly associated with postoperative ΔSE, providing anatomically grounded evidence that refractive outcomes may be linked to globe repositioning within the orbit. Notably, a significant monotonic downward association was also observed between Δexophthalmos and ΔAL, whereby greater reductions in exophthalmos were associated with larger increases in optically measured AL. This finding suggests a potential biomechanical–optical link between orbital decompression-induced structural changes and refractive outcomes, in which Δexophthalmos captures the magnitude of globe translation while ΔAL reflects its optical manifestation as the change in effective optical path length.

Consistent with this interpretation, changes in corneal biomechanical and morphological parameters demonstrated secondary but significant associations with postoperative refractive changes. Parameters reflecting corneal compliance, deformation amplitude, and recovery dynamics suggest that reduced biomechanical stiffness may amplify refractive responses to orbital perturbation [33, 67–69]. Morphological features, including pachymetric distribution and curvature, exerted smaller but meaningful effects, consistent with previous studies linking corneal structural parameters to refractive status [70].

Additionally, eyelid-related changes represent an important but often underappreciated contributor to postoperative refractive behavior. In this study, changes in eyelid position parameters (ΔPFH and ΔMRD1) were significantly associated with postoperative refractive change. Prior studies have demonstrated that eyelid position and morphology can influence corneal shape and optical properties through mechanical interactions with the anterior ocular surface [71]. Changes in eyelid configuration may therefore redistribute external pressure on the corneal surface and further alter corneal morphology and anterior segment geometry [72]. Although direct quantification of eyelid pressure was not performed, the observed correlations supported incorporating eyelid biomechanics into the overall framework explaining how periocular soft tissue dynamics affected postoperative refractive outcomes.

Taken together, these findings support a direct biomechanical–optical coupling framework following orbital decompression. Postoperative ΔSE should be conceptualized as a systems-level response, governed jointly by multiple interacting components, including axial configuration, globe position, corneal structural properties, and eyelid mechanics, which cannot be explained by a single-variable relationship.

Apart from postoperative interactive mechanisms, baseline ocular and orbital characteristics may modulate individual susceptibility to postoperative refractive changes. In the present cohort, greater baseline exophthalmos and larger eyelid aperture were associated with a reduced risk of clinically significant myopic drift. These findings indicate that baseline anterior globe positioning and periocular soft tissue tension may influence external mechanical loading conditions and thereby affect postoperative ocular structural responses. This interpretation is broadly consistent with previous Corvis ST-based studies in TED demonstrating associations between baseline Hertel exophthalmometry values and ocular biomechanical responses following orbital decompression [15], further supporting the concept that orbital anatomy participates in global ocular biomechanical regulation rather than serving merely as a static anatomical descriptor.

Corneal structural architecture also appeared to contribute to susceptibility to postoperative refractive change. Although these concepts were originally developed in the context of corneal ectasia detection, parameters reflecting more homogeneous corneal pachymetric distribution and reduced pachymetric progression were associated with greater refractive stability, consistent with evidence that structural homogeneity facilitates a more even stress distribution and resistance to deformation [73, 74]. Conversely, increased pachymetric gradients and irregular thickness profiles have been linked to biomechanical heterogeneity and reduced structural integrity, which potentially predispose the cornea to greater refractive instability under external biomechanical perturbation [73]. These findings extend the potential relevance of corneal pachymetric architecture beyond ectatic disease to postoperative refractive remodeling following orbital decompression.

Anterior segment geometry and dynamic biomechanical behavior provided complementary insights into susceptibility to postoperative refractive changes. Steeper anterior curvature, greater astigmatism, and increased ACD were associated with greater refractive instability, features that may collectively reflect a more compliant anterior segment configuration. Dynamic corneal response parameters and structural biomechanical indices consistently indicated that baseline biomechanical responsiveness may modulate susceptibility to postoperative refractive change. These observations are in agreement with prior evidence that baseline corneal viscoelastic properties and structural susceptibility influence ocular responses to external mechanical perturbation and deformation propagation [75, 76].

Collectively, these findings support an integrative model in which the baseline orbital configuration, corneal structural properties, and anterior segment biomechanics define an intrinsic biomechanical phenotype that modulates inter-individual susceptibility to postoperative refractive change. This highlights the role of baseline biomechanical set points in shaping refractive outcomes and supports individualized preoperative assessment and risk stratification.

This study had several limitations. Direct measurements of eyelid mechanical pressure were not available, and PFH and MRD1 were used as surrogate indicators; thus, the contribution of eyelid mechanics may have been incompletely captured. Refraction was measured under non-cycloplegic conditions, which may introduce minor variability despite the minimal accommodative influence in adults. The retrospective design and variable follow-up intervals may have introduced inherent heterogeneity in the observation timing and uneven distribution of measurements over time. Continuous-time GEE modeling was applied to accommodate irregular follow-up intervals and intra-subject correlations, but the uneven temporal density of observations may still limit the granularity of the longitudinal trajectory estimation. Additionally, procedure-level findings should be interpreted as exploratory and hypothesis-generating due to modest and uneven sample sizes across subgroups. Finally, the follow-up duration was relatively limited, with observations concentrated in the early postoperative period. Prospective studies with larger cohorts, standardized measurement intervals, and extended follow-up are warranted to validate these findings.

Despite these limitations, the integration of longitudinal modeling, multimodal biometric assessment, and a structured analytical framework provides a coherent mechanistic perspective on postoperative refractive changes following orbital decompression. Future studies incorporating larger cohorts, extended follow-up, and direct biomechanical measurements will further refine individualized risk prediction and help elucidate long-term refractive dynamics. Clinically, these findings support the importance of preoperative risk stratification and postoperative refractive monitoring, with deferral of definitive refractive correction until stabilization, particularly in patients with high visual demands.

## Conclusions

Orbital decompression was associated with dynamic, predominantly spherical, refractive changes that varied with surgical strategy and individual ocular characteristics. Postoperative refractive changes appeared to peak at approximately 2–3 months and partially recovered thereafter, with refractive status gradually approaching relative stabilization over time. ΔAL showed the strongest association with postoperative refractive changes, in conjunction with contributions from ocular positional, eyelid-related, corneal biomechanical, and corneal tomographic changes. These findings highlight postoperative refractive change as a clinically relevant outcome of orbital decompression and support individualized surgical planning, perioperative management, and postoperative refractive monitoring of TED.

## Supplementary Information

Additional file 1. Supplementary methods

Additional file 2. Definitions of the Corvis ST and Pentacam parameters

Additional file 3. Covariate adjustment strategy for main-text analyses and supplementary analyses

Additional file 4. Supplementary methods and results

Additional file 5. Baseline clinical, corneal biomechanical, and morphological characteristics of the cohort

Additional file 6. Standardized multivariable RCS-based GEE analysis of associations between postoperative ocular biometric changes and postoperative ΔSE.

Additional file 7. Non-standardized multivariable RCS-based GEE analysis of associations between postoperative ocular biometric changes and postoperative ΔSE.

Additional file 8. Significant exposure–response associations between postoperative ocular biometric changes and postoperative ΔSE identified from non-standardized RCS-based GEE models. Exposure–response curves for postoperative changes in ocular biometric parameters that showed statistically significant associations with postoperative ΔSE in non-standardized RCS-based GEE models. For clarity of interpretation, all parameters are grouped into three predefined categories according to their clinical and biomechanical characteristics: Group A: clinical variables encompassing orbital structural parameters, inflammatory activity, eyelid position metrics, and AL; Group B: Corvis ST parameters representing corneal biomechanical responses; Group C: corneal morphological variables describing corneal shape and tomographic features. For each panel, associations were further classified according to their functional forms, and key features such as extremum points, were indicated where applicable. AL, axial length; ARTh, Ambrósio’s relational thickness; BAD-D, Belin/Ambrósio Display D index; bIOP, biomechanically corrected intraocular pressure; CAS, Clinical Activity Score; dArc, deformation arc; Db, back elevation deviation; Df, front elevation deviation; GEE, generalized estimating equations; HC, highest concavity; ISV, index of surface variance; MRD1, margin reflex distance 1; PFH, palpebral fissure height; RCS, restricted cubic splines; WEM time, maximum whole eye movement time

Additional file 9. Sensitivity analysis of associations between postoperative ocular biometric changes and postoperative refractive change excluding thyroid function indices.

Additional file 10. Standardized multivariable logistic GEE analysis of the associations between baseline ocular biometric parameters and clinically significant postoperative myopic drift.

Additional file 11. Non-standardized multivariable RCS-based GEE analysis of associations between baseline ocular biometric parameters and postoperative ΔSE.

Additional file 12. Significant exposure–response associations between baseline ocular biometric parameters and postoperative ΔSE identified from non-standardized RCS-based GEE models. Exposure–response curves for baseline ocular biometric parameters that demonstrated statistically significant associations with postoperative ΔSE in non-standardized RCS-based GEE models. Associations are categorized according to their functional patterns, and key features such as extremum points are indicated where applicable. GEE, generalized estimating equations; RCS, restricted cubic splines; SE, spherical equivalent

Additional file 13. Sensitivity analysis of baseline ocular biometric predictors of clinically significant myopic drift excluding thyroid function indices.

## Data Availability

Data supporting the findings of this study are available from the corresponding author upon request.

## Abbreviations

AL: Axial length
AR(1): First-order autoregressive working correlation structure
BCVA: Best-corrected visual acuity
bIOP: Biomechanically corrected intraocular pressure
BMLD: Balanced medial–lateral wall decompression
CAS: Clinical Activity Score
CCT: Central corneal thickness
CI: Confidence interval
D: Diopter
DC: Cylindrical diopter
DS: Spherical diopter
EUGOGO: European Group on Graves’ Orbitopathy
FT3: Free triiodothyronine
FT4: Free thyroxine
GEE: Generalized estimating equations
IQR: Interquartile range
IOP: Intraocular pressure
LWD: Lateral wall decompression
MRD1: Margin reflex distance 1
MWD: Medial wall decompression
OR: Odds ratio
PFH: Palpebral fissure height
RCS: Restricted cubic splines
SD: Standard deviation
SE: Spherical equivalent
StdErr: Standard error
TED: Thyroid eye disease
TSH: Thyroid-stimulating hormone
VIF: Variance inflation factor

## References

[1] Bartalena L, Kahaly GJ, Baldeschi L, Dayan CM, Eckstein A, Marcocci C, et al. The 2021 European Group on Graves’ Orbitopathy (EUGOGO) clinical practice guidelines for the medical management of Graves’ orbitopathy. Eur J Endocrinol. 2021;185(4):G43-67. 34297684 10.1530/EJE-21-0479

[2] Bartalena L, Piantanida E, Gallo D, Lai A, Tanda ML. Epidemiology, natural history, risk factors, and prevention of Graves’ orbitopathy. Front Endocrinol (Lausanne). 2020;11:615993. 33329408 10.3389/fendo.2020.615993 PMC7734282

[3] Burch HB, Perros P, Bednarczuk T, Cooper DS, Dolman PJ, Leung AM, et al. Management of thyroid eye disease: a consensus statement by the American Thyroid Association and the European Thyroid Association. Thyroid. 2022;32(12):1439–70. 36480280 10.1089/thy.2022.0251 PMC9807259

[4] Mourits MP, Bijl H, Altea MA, Baldeschi L, Boboridis K, Currò N, et al. Outcome of orbital decompression for disfiguring proptosis in patients with Graves’ orbitopathy using various surgical procedures. Br J Ophthalmol. 2009;93(11):1518–23. 19028743 10.1136/bjo.2008.149302

[5] Park CY. Factors affecting postoperative satisfaction after presbyopia-correcting intraocular lens. J Clin Med. 2026;15(1):336. 41517585 10.3390/jcm15010336 PMC12786824

[6] Lin FY, Ho RW, Yu HJ, Yang IH, Fang PC, Kuo MT. Impacts and correlations on corneal biomechanics, corneal optical density, and intraocular pressure after cataract surgery. Diagnostics (Basel). 2024;14(14):1557. 39061693 10.3390/diagnostics14141557 PMC11275892

[7] Chandrasekaran S, Petsoglou C, Billson FA, Selva D, Ghabrial R. Refractive change in thyroid eye disease (a neglected clinical sign). Br J Ophthalmol. 2006;90(3):307–9. 16488951 10.1136/bjo.2005.078295 PMC1856938

[8] Kim WS, Chun YS, Cho BY, Lee JK. Biometric and refractive changes after orbital decompression in Korean patients with thyroid-associated orbitopathy. Eye (Lond). 2016;30(3):400–5. 26584795 10.1038/eye.2015.242 PMC4791698

[9] Norris JH, Ross JJ, Kazim M, Selva D, Malhotra R. The effect of orbital decompression surgery on refraction and intraocular pressure in patients with thyroid orbitopathy. Eye (Lond). 2012;26(4):535–43. 22261739 10.1038/eye.2011.362 PMC3325579

[10] Sagili S, Desousa JL, Malhotra R. Intraocular pressure and refractive changes following orbital decompression with intraconal fat excision. Open Ophthalmol J. 2008;2:73–6. 19517037 10.2174/1874364100802010073 PMC2694603

[11] Mombaerts I, Vandelanotte S, Koornneef L. Corneal astigmatism in Graves’ ophthalmopathy. Eye (Lond). 2006;20(4):440–6. 15846381 10.1038/sj.eye.6701898

[12] Alsuhaibani AH, Carter KD, Policeni B, Nerad JA. Orbital volume and eye position changes after balanced orbital decompression. Ophthalmic Plast Reconstr Surg. 2011;27(3):158–63. 20940662 10.1097/IOP.0b013e3181ef72b3

[13] Leite CA, Pereira TS, Chiang J, Moritz RB, Gonçalves ACP, Monteiro MLR. Ocular motility changes after inferomedial wall and balanced medial plus lateral wall orbital decompression in Graves’ orbitopathy: a randomized prospective comparative study. Clinics (Sao Paulo). 2021;76:e2592. 33852655 10.6061/clinics/2021/e2592 PMC8009066

[14] Hsia Y, Wei YH, Liao SL. Changes in ocular biomechanical response parameters and intraocular pressure after surgical treatment for thyroid eye disease. Invest Ophthalmol Vis Sci. 2023;64(10):31. 37494009 10.1167/iovs.64.10.31 PMC10382999

[15] Soleymanzadeh M, Rafizadeh SM, Ghochani G, Mafi AR, Nazari M, Rajabi MT. Biomechanical changes of the cornea after orbital decompression in thyroid-associated orbitopathy measured by Corvis ST. Sci Rep. 2024;14(1):16930. 39043930 10.1038/s41598-024-68081-8 PMC11266539

[16] Otto AJ, Koornneef L, Mourits MP, Deen-van Leeuwen L. Retrobulbar pressures measured during surgical decompression of the orbit. Br J Ophthalmol. 1996;80(12):1042–5. 9059266 10.1136/bjo.80.12.1042 PMC505699

[17] Zhang L, Wang Y, Xie LL, Geng WL, Zuo T. The relationship between corneal biomechanics and corneal shape in normal myopic eyes. J Clin Exp Ophthalmol. 2013;4:1–6.

[18] Bartley GB, Gorman CA. Diagnostic criteria for Graves’ ophthalmopathy. Am J Ophthalmol. 1995;119(6):792–5. 7785696 10.1016/s0002-9394(14)72787-4

[19] McKeag D, Lane C, Lazarus JH, Baldeschi L, Boboridis K, Dickinson AJ, et al. Clinical features of dysthyroid optic neuropathy: a European Group on Graves’ Orbitopathy (EUGOGO) survey. Br J Ophthalmol. 2007;91(4):455–8. 17035276 10.1136/bjo.2006.094607 PMC1994756

[20] Segni M, Bartley GB, Garrity JA, Bergstralh EJ, Gorman CA. Comparability of proptosis measurements by different techniques. Am J Ophthalmol. 2002;133(6):813–8. 12036674 10.1016/s0002-9394(02)01429-0

[21] Ameri H, Fenton S. Comparison of unilateral and simultaneous bilateral measurement of globe position using the Hertel exophthalmometer. Ophthalmic Plast Reconstr Surg. 2004;20(6):448–51. 15599245 10.1097/01.iop.0000143712.42344.8c

[22] Guimarães FC, Cruz AA. Palpebral fissure height and downgaze in patients with Graves upper eyelid retraction and congenital blepharoptosis. Ophthalmology. 1995;102(8):1218–22. 9097750 10.1016/s0161-6420(95)30887-1

[23] Putterman AM. Margin reflex distance (MRD) 1, 2, and 3. Ophthalmic Plast Reconstr Surg. 2012;28(4):308–11. 22785597 10.1097/IOP.0b013e3182523b7f

[24] Boboridis K, Assi A, Indar A, Bunce C, Tyers AG. Repeatability and reproducibility of upper eyelid measurements. Br J Ophthalmol. 2001;85(1):99–101. 11133723 10.1136/bjo.85.1.99 PMC1723668

[25] Padhy D, Bharadwaj SR, Nayak S, Rath S, Das T. Does the accuracy and repeatability of refractive error estimates depend on the measurement principle of autorefractors? Transl Vis Sci Technol. 2021;10(1):2. 33505769 10.1167/tvst.10.1.2 PMC7794271

[26] Bailey MD, Twa MD, Mitchell GL, Dhaliwal DK, Jones LA, McMahon TT. Repeatability of autorefraction and axial length measurements after LASIK. J Cataract Refract Surg. 2005;31(5):1025–34. 15975474 10.1016/j.jcrs.2004.12.040

[27] Petrovic Jurcevic J, Jurcevic M, Jagic M, Jazbec A, Mandic K, Juri Mandic J. Influence of clinically active Graves’ ophthalmopathy on spherical equivalent and visual acuity. Clin Ophthalmol. 2022;16:2353–61. 35924183 10.2147/OPTH.S369677 PMC9342880

[28] Khorrami-Nejad M, Khodair AM, Khodaparast M, Babapour Mofrad F, Dehghanian NF. Comparison of ocular ultrasonic and optical biometry devices in the different quality measurements. J Optom. 2023;16(4):284–95. 37567838 10.1016/j.optom.2023.05.001 PMC10518768

[29] Wiseman SJ, Tatham AJ, Meijboom R, Terrera GM, Hamid C, Doubal FN, et al. Measuring axial length from magnetic resonance brain imaging. BMC Ophthalmol. 2022;22(1):54. 35123441 10.1186/s12886-022-02289-y PMC8817515

[30] Drexler W, Findl O, Menapace R, Rainer G, Vass C, Hitzenberger CK, et al. Partial coherence interferometry: a novel approach to biometry in cataract surgery. Am J Ophthalmol. 1998;126(4):524–34. 9780097 10.1016/s0002-9394(98)00113-5

[31] Norrby S. Sources of error in intraocular lens power calculation. J Cataract Refract Surg. 2008;34(3):368–76. 18299059 10.1016/j.jcrs.2007.10.031

[32] Vinciguerra R, Elsheikh A, Roberts CJ, Ambrósio R Jr, Kang DS, Lopes BT, et al. Influence of pachymetry and intraocular pressure on dynamic corneal response parameters in healthy patients. J Refract Surg. 2016;32(8):550–61. 27505316 10.3928/1081597X-20160524-01

[33] Vinciguerra R, Ambrósio R Jr, Elsheikh A, Roberts CJ, Lopes BT, Morenghi E, et al. Detection of keratoconus with a new biomechanical index. J Refract Surg. 2016;32(12):803–10. 27930790 10.3928/1081597X-20160629-01

[34] Khoramnia R, Rabsilber TM, Auffarth GU. Central and peripheral pachymetry measurements according to age using the Pentacam rotating Scheimpflug camera. J Cataract Refract Surg. 2007;33(5):830–6. 17466857 10.1016/j.jcrs.2006.12.025

[35] Kanclerz P, Khoramnia R, Wang X. Current developments in corneal topography and tomography. Diagnostics (Basel). 2021;11(8):1466. 34441401 10.3390/diagnostics11081466 PMC8392046

[36] Belin MW, Khachikian SS, Ambrósio R Jr. Elevation-based corneal tomography. 2nd ed. Panama City: Jaypee; 2012.

[37] Pentacam AXL | OCULUS. https://www.oculus.de/en/products/pentacam-axl/?pentacam-family. Accessed 25 Apr 2024.

[38] Corvis® ST—Evaluation of corneal biomechanical response, tonometry and pachymetry | OCULUS. https://www.oculus.de/en/products/corvis-st/. Accessed 25 Apr 2024.

[39] Yu A, Zhao W, Savini G, Huang Z, Bao F, Lu W, et al. Evaluation of central corneal thickness using Corvis ST and comparison with Pentacam and ultrasound pachymetry in normal eyes. J Ophthalmol. 2015;2015:767012. 26697213 10.1155/2015/767012 PMC4678087

[40] Rajabi S, Asharlous A, Riazi A, Khabazkhoob M, Moalej A. Differences and limits of agreement among Pentacam, Corvis ST, and IOLMaster 700 for central corneal thickness measurements. J Curr Ophthalmol. 2022;34(1):44–9. 35620377 10.4103/joco.joco_96_21 PMC9128434

[41] Li HG, Chen YH, Lin F, Li SY, Liu QH, Yin CG, et al. Agreement of intraocular pressure measurement with Corvis ST, non-contact tonometer, and Goldmann applanation tonometer in children with ocular hypertension and related factors. Int J Ophthalmol. 2023;16(10):1601–7. 37854370 10.18240/ijo.2023.10.07 PMC10559031

[42] Karmiris E, Tsiogka A, Stavrakas P, Tsiripidis K, Papakonstantinou E, Chalkiadaki E. Comparison of intraocular pressure measurements obtained by Goldmann applanation tonometer, Corvis ST and a conventional non-contact airpuff tonometer in eyes with previous myopic refractive surgery and correlation with corneal biomechanical parameters. Int Ophthalmol. 2025;45(1):232. 40478442 10.1007/s10792-025-03598-z

[43] Jefferis JM, Jones RK, Currie ZI, Tan JH, Salvi SM. Orbital decompression for thyroid eye disease: methods, outcomes, and complications. Eye (Lond). 2018;32(3):626–36. 29243735 10.1038/eye.2017.260 PMC5848288

[44] Braun TL, Bhadkamkar MA, Jubbal KT, Weber AC, Marx DP. Orbital decompression for thyroid eye disease. Semin Plast Surg. 2017;31(1):40–5. 28255288 10.1055/s-0037-1598192 PMC5330789

[45] Durrleman S, Simon R. Flexible regression models with cubic splines. Stat Med. 1989;8(5):551–61. 2657958 10.1002/sim.4780080504

[46] Harrell FE. Regression modeling strategies: with applications to linear models, logistic and ordinal regression, and survival analysis. 2nd ed. Cham: Springer; 2015.

[47] Perperoglou A, Sauerbrei W, Abrahamowicz M, Schmid M. A review of spline function procedures in R. BMC Med Res Methodol. 2019;19(1):46. 30841848 10.1186/s12874-019-0666-3 PMC6402144

[48] Liang KY, Zeger SL. Longitudinal data analysis using generalized linear models. Biometrika. 1986;73:13–22.

[49] Zeger SL, Liang KY, Albert PS. Models for longitudinal data: a generalized estimating equation approach. Biometrics. 1988;44(4):1049–60. 3233245

[50] Bullimore MA, Fusaro RE, Adams CW. The repeatability of automated and clinician refraction. Optom Vis Sci. 1998;75(8):617–22. 9734807 10.1097/00006324-199808000-00028

[51] Zhou J, Gu W, Gao Y, Wang W, Zhang F. Survival analysis of myopic regression after small incision lenticule extraction and femtosecond laser-assisted laser in situ keratomileusis for low to moderate myopia. Eye Vis (Lond). 2022;9(1):28. 35909114 10.1186/s40662-022-00300-7 PMC9341088

[52] Glymour MM, Weuve J, Berkman LF, Kawachi I, Robins JM. When is baseline adjustment useful in analyses of change? An example with education and cognitive change. Am J Epidemiol. 2005;162(3):267–78. 15987729 10.1093/aje/kwi187

[53] Robins JM, Hernán MA, Brumback B. Marginal structural models and causal inference in epidemiology. Epidemiology. 2000;11(5):550–60. 10955408 10.1097/00001648-200009000-00011

[54] VanderWeele TJ. Explanation in causal inference: methods for mediation and interaction. Oxford: Oxford University Press; 2015.

[55] Kim KW, Byun JS, Lee JK. Surgical effects of various orbital decompression methods in thyroid-associated orbitopathy: computed tomography-based comparative analysis. J Craniomaxillofac Surg. 2014;42(7):1286–91. 24793198 10.1016/j.jcms.2014.03.011

[56] Cohen J. Statistical power analysis for the behavioral sciences. 2nd ed. New York: Routledge; 1988.

[57] Gelman A. Scaling regression inputs by dividing by two standard deviations. Stat Med. 2008;27(15):2865–73. 17960576 10.1002/sim.3107

[58] Marquaridt DW. Generalized inverses, ridge regression, biased linear estimation, and nonlinear estimation. Technometrics. 1970;12:591–612.

[59] Faul F, Erdfelder E, Buchner A, Lang AG. Statistical power analyses using G*Power 3.1: tests for correlation and regression analyses. Behav Res Methods. 2009;41(4):1149–60. 19897823 10.3758/BRM.41.4.1149

[60] R Core Team. R: a language and environment for statistical computing. Vienna: R Foundation for Statistical Computing; 2024. https://www.R-project.org/. Accessed 30 Apr 2024.

[61] Ishii R, Ohigashi T, Maruo K, Gosho M. Geessbin: an R package for analyzing small-sample binary data using modified generalized estimating equations with bias-adjusted covariance estimators. BMC Med Res Methodol. 2024;24(1):277. 39538119 10.1186/s12874-024-02368-2 PMC11558877

[62] Fox J, Weisberg S. An R companion to applied regression, third edition. Thousand Oaks CA: Sage; 2019.

[63] Wickham H. ggplot2: Elegant Graphics for Data Analysis. Springer-Verlag New York; 2016.

[64] Park HH, Chun YS, Moon NJ, Kim JT, Park SJ, Lee JK. Change in eyelid parameters after orbital decompression in thyroid-associated orbitopathy. Eye (Lond). 2018;32(6):1036–41. 29391576 10.1038/s41433-018-0022-6 PMC5997754

[65] Cho RI, Elner VM, Nelson CC, Frueh BR. The effect of orbital decompression surgery on lid retraction in thyroid eye disease. Ophthalmic Plast Reconstr Surg. 2011;27(6):436–8. 21785378 10.1097/IOP.0b013e3182232465

[66] Aramberri J, Hoffer KJ, Olsen T, Savini G, Shammas HJ. Axial length measurement. In: Aramberri J, Hoffer KJ, Olsen T, Savini G, Shammas HJ, editors. Intraocular lens calculations. Cham: Springer; 2024.

[67] Sinha Roy A, Dupps WJ Jr. Effects of altered corneal stiffness on native and postoperative LASIK corneal biomechanical behavior: a whole-eye finite element analysis. J Refract Surg. 2009;25(10):875–87. 19835328 10.3928/1081597X-20090917-09

[68] Damgaard IB, Reffat M, Hjortdal J. Review of corneal biomechanical properties following LASIK and SMILE for myopia and myopic astigmatism. Open Ophthalmol J. 2018;12:164–74. 30123381 10.2174/1874364101812010164 PMC6062908

[69] Li DL, Liu MX, Yin ZJ, Li YZ, Ma R, Zheng YJ, et al. Refractive associations with corneal biomechanical properties among young adults: a population-based Corvis ST study. Graefes Arch Clin Exp Ophthalmol. 2024;262(1):121–32. 37401934 10.1007/s00417-023-06164-4

[70] Rosenblatt A, Mimouni M, Sela T, Munzer G, Varssano D, Sorkin N. Correlation between refractive state, corneal thickness, and keratometry in ametropic patients. Eur J Ophthalmol. 2020;30(5):891–6. 31055942 10.1177/1120672119845609

[71] Read SA, Collins MJ, Carney LG. The influence of eyelid morphology on normal corneal shape. Invest Ophthalmol Vis Sci. 2007;48(1):112–9. 17197524 10.1167/iovs.06-0675

[72] Shaw AJ, Collins MJ, Davis BA, Carney LG. Corneal refractive changes due to short-term eyelid pressure in downward gaze. J Cataract Refract Surg. 2008;34(9):1546–53. 18721718 10.1016/j.jcrs.2008.05.047

[73] Ambrósio R Jr, Lopes BT, Faria-Correia F, Salomão MQ, Bühren J, Roberts CJ, et al. Integration of Scheimpflug-based corneal tomography and biomechanical assessments for enhancing ectasia detection. J Refract Surg. 2017;33(7):434–43. 28681902 10.3928/1081597X-20170426-02

[74] Roberts CJ, Mahmoud AM, Bons JP, Hossain A, Elsheikh A, Vinciguerra R, et al. Introduction of two novel stiffness parameters and interpretation of air puff-induced biomechanical deformation parameters with a dynamic scheimpflug analyzer. J Refract Surg. 2017;33(4):266–73. 28407167 10.3928/1081597X-20161221-03

[75] Roberts CJ, Dupps WJ Jr. Biomechanics of corneal ectasia and biomechanical treatments. J Cataract Refract Surg. 2014;40(6):991–8. 24774009 10.1016/j.jcrs.2014.04.013 PMC4850839

[76] Roberts CJ. Concepts and misconceptions in corneal biomechanics. J Cataract Refract Surg. 2014;40(6):862–9. 24857435 10.1016/j.jcrs.2014.04.019

