# Supplementary material for: Longitudinal refractive changes following orbital decompression in thyroid eye disease: dominant role of axial configuration and globe position with contributions from surgical technique and corneal biomechanics
Source: Eye Vis (Lond). 2026 Jul 22;13:32. doi: 10.1186/s40662-026-00499-9 (PMC13390221; doi:10.1186/s40662-026-00499-9)
Supplement: Supplementary file 1 — Additional file 1. Supplementary methods [file 40662_2026_499_MOESM1_ESM.docx]

**Additional File 1: Supplementary Methods**

**Supplementary Methods S1. Detailed participant selection and exclusion criteria**

Surgical exclusion criteria included: (1) abnormal thyroid function, defined as serum free triiodothyronine (FT3), free thyroxine (FT4), or thyroid-stimulating hormone (TSH) levels outside institutional reference ranges within 6 months prior to surgery; (2) uncontrolled systemic diseases, including hypertension, diabetes mellitus, renal disease, or cardiovascular disorders; (3) pregnancy or lactation; and (4) diagnosed psychiatric disorders, including mood, anxiety, or psychotic disorders.

For inclusion in the analytical dataset, eyes were further excluded if they met any of the following criteria:

1. Eyes with no postoperative follow-up examinations within the study period;
2. Eyes that developed corneal erosion (≥ grade 2) or dysthyroid optic neuropathy during follow-up;
3. Eyes with incomplete data for any key parameter required for analysis;
4. Age < 18 years or > 60 years;
5. History of steroid therapy (local injection, oral, or intravenous) or immunosuppressive treatment within 3 months before surgery;
6. History of orbital or ocular trauma or surgery;
7. Pre-existing corneal pathology, including inflammation, thinning, ulceration, scarring, or corneal erosion (≥ grade 2, Oxford scheme);
8. Ophthalmic comorbidities, including glaucoma, uveitis, diabetic retinopathy, vitreoretinopathy, or maculopathy;
9. Soft contact lens use within 2 weeks or rigid contact lens use within 3 weeks before surgery;
10. Best-corrected visual acuity (BCVA) < 0.2 on the decimal scale, to ensure adequate fixation and reliable acquisition of refractive and corneal imaging data;
11. Extreme refractive errors, including spherical equivalent (SE) < −6.00 D or > +2.00 D, absolute cylindrical diopter (DC) > 3.00 D, or Pentacam-measured corneal astigmatism > 3.00 D, to reduce physiological heterogeneity and ensure measurement reliability.

**Supplementary Methods S2. Standardized surgical technique for bony orbital decompression with fat excision**

All surgeries were performed under general anesthesia using standardized preservation-based techniques, with patients in the supine position following standard sterile preparation.

1. **Lateral wall decompression (transcutaneous preservation-based approach)**

A short lateral horizontal skin incision (1–2 cm) was made along the lateral orbital rim. The lateral canthus was preserved, and dissection was confined to the lateral orbital rim and bony wall. No upper eyelid crease incisions or routine lateral canthotomy/canthopexy were performed. Lateral canthal tendons and tarsoligamentous support structures were preserved throughout the procedure.

Blunt dissection exposed the lateral orbital rim, and the periosteum was incised using monopolar electrocautery. The lateral orbital rim was osteotomized using an oscillating saw, and the bone flap was temporarily removed.

The lacrimal gland fossa was enlarged using a drill. Deep portions of the greater wing of the sphenoid between the superior and inferior orbital fissures were removed to allow adequate orbital expansion while avoiding dural injury.

A tongue-shaped segment of lateral wall periosteum was excised for histopathological analysis. Blunt intraorbital dissection facilitated herniation of orbital contents into the temporal fossa. Inferolateral orbital fat was partially excised as required.

1. **Medial wall decompression (transconjunctival approach)**

Medial wall decompression was performed via a transconjunctival approach that avoided external skin incisions and eyelid-support structures.

An arcuate conjunctival incision was made near the medial caruncle. The anterior and posterior ethmoidal arteries were cauterized. Portions of the lamina papyracea and inferomedial orbital angle were removed using rongeurs, permitting prolapse of orbital tissues into the ethmoid sinus.

Blunt dissection allowed adequate decompression of the medial orbital compartment. Appropriate amounts of medial orbital fat were excised when necessary.

1. **Closure and reconstruction**

After confirming hemostasis, the lateral wall bone flap was trimmed and repositioned using titanium plates and screws.

Periosteum and subcutaneous tissues were closed using absorbable sutures. The skin incision was closed with interrupted sutures. The conjunctival incision was closed with absorbable stitches.

A rubber drain was placed in the temporal fossa. Antibiotic ophthalmic ointment was applied and the operated eye was pressure-bandaged.

1. **Orbital fat volume measurement**

Excised orbital fat was carefully collected intraoperatively, and its volume was measured using a calibrated syringe.
