# Supplementary material for: Longitudinal refractive changes following orbital decompression in thyroid eye disease: dominant role of axial configuration and globe position with contributions from surgical technique and corneal biomechanics
Source: Eye Vis (Lond). 2026 Jul 22;13:32. doi: 10.1186/s40662-026-00499-9 (PMC13390221; doi:10.1186/s40662-026-00499-9)
Supplement: Supplementary file 4 — Additional file 4. Supplementary methods and results [file 40662_2026_499_MOESM4_ESM.docx]

**Additional File 4. Supplementary Methods and Results**

**Supplementary Analysis S1. Procedure-level analysis of postoperative refractive change**

**Supplementary Methods S1**

To complement the primary analyses based on decompression extent, additional exploratory analyses were conducted to evaluate postoperative refractive change (ΔSE) across individual surgical procedures, including medial wall decompression (MWD), lateral wall decompression (LWD), and balanced medial–lateral wall decompression (BMLD), as defined in the main text.

Generalized estimating equations (GEE) models with a Gaussian family and identity link were applied to estimate population-averaged associations between surgical procedures and postoperative ΔSE across repeated follow-up visits. To improve interpretability of procedure-level associations, two complementary model specifications were applied. First, models without adjustment for postoperative exophthalmos reduction (Δexophthalmos) were used to estimate the overall associations between surgical procedures and ΔSE as observed in clinical practice. Second, models additionally adjusting for Δexophthalmos were fitted to account for variability in decompression extent, allowing separation of procedure-related differences from those attributable to decompression extent. For each specification, two model structures were implemented. First, main-effects models included surgical procedure as a categorical predictor and follow-up time (months) as a continuous variable to estimate average differences in ΔSE across procedures. Second, interaction models further incorporated a procedure × follow-up time term to evaluate differences in temporal patterns of postoperative refractive change. Procedure-specific temporal slopes were derived by combining the interaction term with the main time coefficient.

Follow-up time was modeled as a linear continuous variable to maintain consistency with the primary analyses. MWD was specified as the reference category. Pairwise comparisons not directly represented by the reference category were obtained using linear contrasts of fitted coefficients. All model specifications, including the working correlation structure, robust variance estimation, and covariate adjustment framework, were identical to those described in the primary analyses.

**Supplementary Results S1**

**1.1 Overall clinical effects of surgical procedures**

## When evaluated as overall procedural effects in routine clinical practice, individual decompression procedures demonstrated clear heterogeneity in postoperative refractive change, both in magnitude and temporal progression.

In main-effects GEE analyses (Supplementary Table S1a), BMLD was associated with a markedly greater average myopic shift (β = −0.340 D, *P* < 0.001) compared with MWD, while LWD also demonstrated a smaller but statistically significant increase in myopic shift relative to MWD (β = −0.122 D, *P* = 0.031). Pairwise contrasts further confirmed a significantly smaller average myopic shift in LWD than in BMLD (β = 0.218 D, *P* < 0.001, Supplementary Table S1d). Follow-up time remained significantly associated with cumulative myopic progression (β = −0.052 D/month, *P* < 0.001, Supplementary Table S1a).

Temporal trajectory analyses demonstrated procedure-specific differences in progression dynamics (Supplementary Table S1b). Relative to MWD, BMLD exhibited significantly faster myopic progression (β = −0.245 D/month, *P* < 0.001), whereas the difference between LWD and MWD showed only a borderline trend (β = −0.125 D/month, *P* = 0.066). The difference in progression rate between BMLD and LWD was also numerically apparent but did not reach statistical significance (β = 0.120 D/month, *P* = 0.067, Supplementary Table S1d).

Slope estimates showed a graded pattern across procedures (Supplementary Table S1c). MWD showed mild progression (slope = −0.052 D/month), LWD exhibited intermediate myopic progression (slope = −0.178 D/month), and BMLD demonstrated the steepest myopic progression (slope = −0.297 D/month).

Notably, due to modest and uneven sample sizes across subgroups, procedure-level findings should be interpreted as exploratory and hypothesis-generating.

Collectively, these findings indicate that BMLD was associated with the largest magnitude and fastest progression of myopic shift, LWD with intermediate effects, and MWD with the least pronounced refractive alterations.

**1.2 Procedure-specific effects independent of decompression extent**

After adjustment for Δexophthalmos, the magnitude of between-procedure differences in population-average ΔSE was attenuated but remained partially significant (Supplementary Table S1a). BMLD continued to demonstrate a significantly greater average myopic shift relative to MWD (β = −0.294 D, *P* < 0.001), and pairwise contrasts confirmed a significantly smaller myopic shift in LWD compared with BMLD (β = 0.218 D, *P* < 0.001, Supplementary Table S1d), whereas the difference between LWD and MWD was no longer statistically significant (β = −0.077 D, *P* = 0.166). The temporal effect of follow-up time also disappeared after adjustment (β = 0.009 D/month, *P* = 0.531, Supplementary Table S1a).

In the adjusted interaction model, procedure-specific differences in progression dynamics persisted (Supplementary Table S1b). BMLD remained associated with significantly steeper myopic progression relative to MWD (β = −0.254 D/month, *P* < 0.001), with an effect size comparable to the unadjusted model (β = −0.245 D/month). Notably, LWD now demonstrated a statistically significant faster progression rate relative to MWD (β = −0.149 D/month, *P* = 0.019), whereas this association had been only borderline significant before adjustment (*P* = 0.066). The difference in progression rate between BMLD and LWD remained statistically non-significant (β = 0.105 D/month, *P* = 0.095, Supplementary Table S1d).

Slope analyses reinforced a graded pattern across procedures (Supplementary Table S1c). After adjustment, MWD exhibited essentially stable refractive status with a slight hyperopic tendency (slope = 0.009 D/month vs. −0.052 D/month previously). LWD maintained intermediate progression (slope = −0.140 D/month vs. slope = −0.178 D/month previously), while BMLD continued to show the fastest myopic progression (slope = −0.245 D/month vs. slope = −0.297 D/month previously).

Taken together, the attenuation of average effects after adjustment, coupled with persistence of progression-rate differences, suggests that postoperative refractive change is jointly shaped by decompression extent and the intrinsic properties of surgical procedures.

**Supplementary Table S1. Procedure-level effects of surgical procedures on postoperative refractive change estimated under complementary frameworks.**

| **a Main-effects GEE model** | | | | | | | | |
| --- | --- | --- | --- | --- | --- | --- | --- | --- |
| **Variable** | **Overall effects** | | | | **Magnitude-adjusted effects** | | | |
|  | **β (D)** | **StdErr** | ***P* value** | **95% CI** | **β (D)** | **StdErr** | ***P* value** | **95% CI** |
| BMLD vs. MWD | −0.340 | 0.061 | **< 0.001** | −0.459 to −0.221 | −0.294 | 0.058 | **< 0.001** | −0.407 to −0.181 |
| LWD vs. MWD | −0.122 | 0.057 | **0.031** | −0.233 to −0.011 | −0.077 | 0.055 | 0.166 | −0.185 to 0.032 |
| Follow-up time (months) | −0.052 | 0.012 | **< 0.001** | −0.075 to −0.030 | 0.009 | 0.015 | 0.531 | −0.020 to 0.038 |
| **b Interaction GEE model** | | | | | | | | |
| **Variable** | **Overall effects** | | | | **Magnitude-adjusted effects** | | | |
|  | **β (D/month)** | **StdErr** | ***P* value** | **95% CI** | **β (D/month)** | **StdErr** | P **value** | **95% CI** |
| BMLD × Time vs. MWD × Time | −0.245 | 0.063 | **< 0.001** | −0.369 to −0.122 | −0.254 | 0.055 | **< 0.001** | −0.362 to −0.146 |
| LWD × Time vs. MWD × Time | −0.125 | 0.068 | 0.066 | −0.259 to 0.008 | −0.149 | 0.063 | **0.019** | −0.273 to −0.025 |
| **c Procedure-specific temporal slopes** | | | | | | | | |
| **Surgical procedure** | **Overall effects** | | | | **Magnitude-adjusted effects** | | | |
|  | **Slope (D/month)** | | | | **Slope (D/month)** | | | |
| MWD | −0.052 | | | | 0.009 | | | |
| LWD | −0.178 | | | | −0.140 | | | |
| BMLD | −0.297 | | | | −0.245 | | | |
| **d Pairwise contrasts between surgical procedures** | | | | | | | | |
| **Comparison** | **Overall effects** | | | | **Magnitude-adjusted effects** | | | |
|  | **β** | **StdErr** | ***P* value** | **95% CI** | **β** | **StdErr** | ***P* value** | **95% CI** |
| LWD vs. BMLD | 0.218 | 0.040 | **< 0.001** | 0.140 to 0.296 | 0.218 | 0.041 | **< 0.001** | 0.138 to 0.297 |
| LWD × Time vs. BMLD × Time | 0.120 | 0.065 | 0.067 | −0.008 to 0.248 | 0.105 | 0.063 | 0.095 | −0.018 to 0.229 |

AL = axial length; bIOP = biomechanically corrected intraocular pressure; BMLD = balanced medial–lateral wall decompression; CAS = Clinical Activity Score; CCT = central corneal thickness; CI = confidence interval; GEE = generalized estimating equations; LWD = lateral wall decompression; MWD = medial wall decompression; StdErr = standard error; β = regression coefficient

Surgical procedures were analyzed at the individual procedure level, with results presented under overall and magnitude-adjusted analytical frameworks. (**a**) Main-effects GEE models estimating the independent population-averaged effects of surgical procedure and follow-up time on postoperative ΔSE. (**b**) Interaction GEE models evaluating differential temporal trajectories of postoperative ΔSE through inclusion of the surgical procedure × follow-up time interaction term. (**c**) Procedure-specific temporal slopes represent the estimated monthly rates of refractive change within each subgroup and were derived by summing the main-effects time coefficient and the interaction term. (**d**) Pairwise contrasts between surgical procedures were obtained using linear combinations of fitted coefficients, enabling direct pairwise comparisons between non-reference procedures. The MWD group served as the reference category for all models. All models were adjusted for age, sex, baseline ocular parameters (AL, CCT, bIOP, CAS, and exophthalmos), thyroid function indices, and orbital fat removal volume. Postoperative follow-up time was incorporated as an explicitly modeled temporal predictor. Δexophthalmos was additionally adjusted in the magnitude-adjusted analytical framework but not in the overall analytical framework. Bold values indicate statistical significance (*P* < 0.05).

**Supplementary Analysis S2. Investigation of exposure–response relationships between postoperative ocular biometric changes and postoperative refractive change**

**Supplementary Methods S2**

To further explore exposure–response relationships between postoperative ocular biometric changes and postoperative ΔSE (continuous outcome), non-standardized multivariable GEE models were fitted with a Gaussian family and identity link. Each Δparameter was analyzed in a separate model to reduce collinearity, with adjustment for clinically relevant confounders, including age, sex, baseline ocular parameters (AL, CCT, bIOP, CAS), thyroid function indices (FT3, FT4, TSH), postoperative follow-up time, orbital fat removal volume, ΔPFH and ΔMRD1.

An autoregressive working correlation structure of order 1 [AR(1)] was adopted to account for within-subject dependence, and robust (sandwich) variance estimators were used to ensure valid statistical inference. Restricted cubic splines (RCS) with three knots at the 10th, 50th, and 90th percentiles were used to evaluate potential nonlinear associations, with overall effects assessed using joint Wald tests. When the nonlinear term was not statistically significant or spline estimation was unstable due to limited variability, linear models were applied. Exposure–response relationships were visualized using model-based smooth curves and classified as monotonic, U-shaped, or inverted U-shaped, with extremum points defined where the first derivative of the spline function equaled zero.

**Supplementary Results S2**

Postoperative changes in ocular biometric parameters exhibited heterogeneous patterns of associations with postoperative ΔSE, including linear, monotonic nonlinear, U-shaped, and inverted U-shaped patterns. Complete results are presented in Additional File 7, and significant associations are depicted in Additional File 8.

Among clinical variables, ΔCAS (*P* = 0.037), Δexophthalmos (*P* = 0.006), and ΔPFH (*P* = 0.011) demonstrated U-shaped relationships with postoperative ΔSE, indicating that intermediate postoperative changes were associated with the greatest myopic drift, whereas smaller or larger changes corresponded to reduced myopic shift or relative hyperopic change. In contrast, ΔMRD1 showed a monotonic upward association (*P* = 0.001), suggesting a consistent positive relationship with ΔSE. ΔAL exhibited an inverted U-shaped association with ΔSE (*P* < 0.001). Intermediate ΔAL values were associated with minimal myopic shift, whereas both smaller and larger deviations were linked to greater myopic drift.

Corneal biomechanical parameters showed heterogeneous associations with postoperative ΔSE. Among A1-phase indices, ΔA1 time demonstrated an inverted U-shaped relationship with ΔSE (*P* = 0.016), while ΔA1 length (*P* = 0.009) showed a U-shaped association. ΔA1 dArc length displayed a monotonic downward association (*P* = 0.010). For A2-phase parameters, ΔA2 deflection area showed a U-shaped relationship with ΔSE (*P* < 0.001).

Highest-concavity (HC) phase parameters consistently exhibited inverted U-shaped associations. Specifically, ΔHC deflection amplitude (*P* < 0.001), ΔHC deflection length (*P* = 0.013), ΔHC dArc length (*P* < 0.001), and ΔHC deflection area (*P* < 0.001) all demonstrated inverted U-shaped patterns.

Among limit indices, ΔWEM time showed a monotonic downward association (*P* = 0.006). ΔDeflection amplitude max (*P* < 0.001), ΔdArc length max (*P* < 0.001), and Δpeak distance (*P* < 0.001) all exhibited inverted U-shaped relationships with ΔSE. For integrated biomechanical parameters, ΔbIOP showed a monotonic upward trend (*P* = 0.008), whereas ΔPachySlope demonstrated an inverted U-shaped association (*P* < 0.001). ΔARTh showed a monotonic upward association (*P* < 0.001).

Corneal morphological parameters also showed significant nonlinear relationships. On the anterior surface, Δanterior steep K (*P* = 0.034) and Δanterior astigmatism (*P* < 0.001) exhibited inverted U-shaped associations with ΔSE, whereas Δanterior Q value showed a monotonic downward trend (*P* = 0.033). Among posterior corneal parameters, Δposterior steep K exhibited a significant inverted U-shaped association with ΔSE (*P* = 0.004). For parameters with insufficient variability to support spline modeling, linear analyses were performed, demonstrating a significant linear association between Δposterior flat K and ΔSE (*P* = 0.010), whereas Δposterior mean K and Δposterior astigmatism were not significantly associated with ΔSE (*P* > 0.05). BAD-related indices, including ΔDf (*P* < 0.001), ΔDb (*P* < 0.001), and ΔBAD-D (*P* = 0.033), all demonstrated inverted U-shaped relationships with postoperative ΔSE. Similarly, anterior corneal irregularity index ΔISV showed an inverted U-shaped association (*P* = 0.022).

Overall, postoperative ΔSE was influenced by a complex interplay of postoperative ocular biometric changes across axial, ocular positional, eyelid-related, corneal biomechanical, and corneal tomographic domains.

**Supplementary Analysis S3. Sensitivity analysis of associations between postoperative ocular biometric changes and postoperative refractive change excluding thyroid function indices**

**Supplementary Methods S3**

To assess the robustness of the primary findings, sensitivity analyses were performed by excluding thyroid function indices (FT3, FT4, and TSH) from the covariate adjustment set using the same standardized spline-based GEE framework. All model specifications, including working correlation structure, robust variance estimation, and RCS modeling, were identical to the primary analysis.

**Supplementary Results S3**

Overall, the hierarchical structure and relative contributions of postoperative ocular biometric parameters to ΔSE were largely preserved. Complete results are provided in Additional File 9, and significant associations are depicted in Supplementary Fig. S3.

Consistent with primary analyses, ΔAL exhibited the strongest association with ΔSE, with a slightly larger standardized nonlinear effect (|adjusted RCS β| = 1.153, *P* < 0.001), confirming its dominant role in postoperative refractive change. The nonlinear inverted U-shaped relationship and extremum location were similar to those observed in the main model.

Ocular positional and eyelid-related variables followed patterns consistent with the primary analyses. Δexophthalmos (|adjusted RCS β| = 0.211, *P* = 0.009) and ΔMRD1 (|adjusted RCS β| = 0.079, *P* = 0.013) demonstrated significant nonlinear associations with ΔSE, with both curve shapes and effect sizes similar to the main model. Although no longer statistically significant, both ΔPFH and ΔCAS (an inflammatory activity index) showed only modest changes in curve shapes and effect sizes compared with the primary analyses.

Across corneal biomechanical parameters, overall patterns and effect sizes remained stable. Key HC-derived indices continued to show inverted U-shaped relationships. Except for ΔA1 time and ΔA1 deflection area, A1- and A2-related parameters showed trends consistent with the primary analyses, with minor changes in effect sizes and statistical significance. Limit indices, including Δdeflection amplitude max and Δpeak distance, as well as integrated parameters such as ΔPachySlope, remained significantly correlated with ΔSE (*P* < 0.001) with largely preserved effect sizes, supporting their consistent contribution to postoperative refractive change.

Most corneal morphological parameters exhibited trends consistent with those of the primary analyses. Except for Δanterior flat K and Δposterior Q value, anterior and posterior corneal curvature parameters displayed association patterns matching the primary analysis findings. Similarly, BAD-related indices including ΔDf, ΔDb, and ΔBAD-D retained their statistically significant inverted U-shaped associations with ΔSE (all *P* < 0.001), further supporting their contribution to postoperative refractive change.

Collectively, these findings indicate that thyroid function indices did not materially alter the observed associations. Their inclusion in the primary models was a conservative adjustment strategy, ensuring comprehensive control for systemic disease-related variability without altering the substantive conclusions.

**Supplementary Fig. S3. Sensitivity analysis of associations between postoperative ocular biometric parameter changes and postoperative refractive change.** Hierarchical ranking of postoperative ocular biometric parameters based on absolute adjusted RCS regression coefficients derived from multivariable GEE models. Models were adjusted for age, sex, baseline ocular parameters (AL, CCT, bIOP, CAS), postoperative follow-up time, orbital fat removal volume, ΔPFH and ΔMRD1. Parameters are color-coded according to predefined effect-size tiers. AL, axial length; ARTh, Ambrósio’s relational thickness (horizontal); BAD-D, Belin/Ambrósio Display D index; bIOP, biomechanically corrected intraocular pressure; CAS, Clinical Activity Score; CCT, central corneal thickness; Db, back elevation deviation; Df, front elevation deviation; ISV, index of surface variance; K, keratometry; Kmax, maximum anterior corneal keratometry; MRD1, margin reflex distance 1; PFH, palpebral fissure height; RCS, restricted cubic splines; Rmin, minimum Radius of Curvature; SE, spherical equivalent; WEM time, maximum whole eye movement time


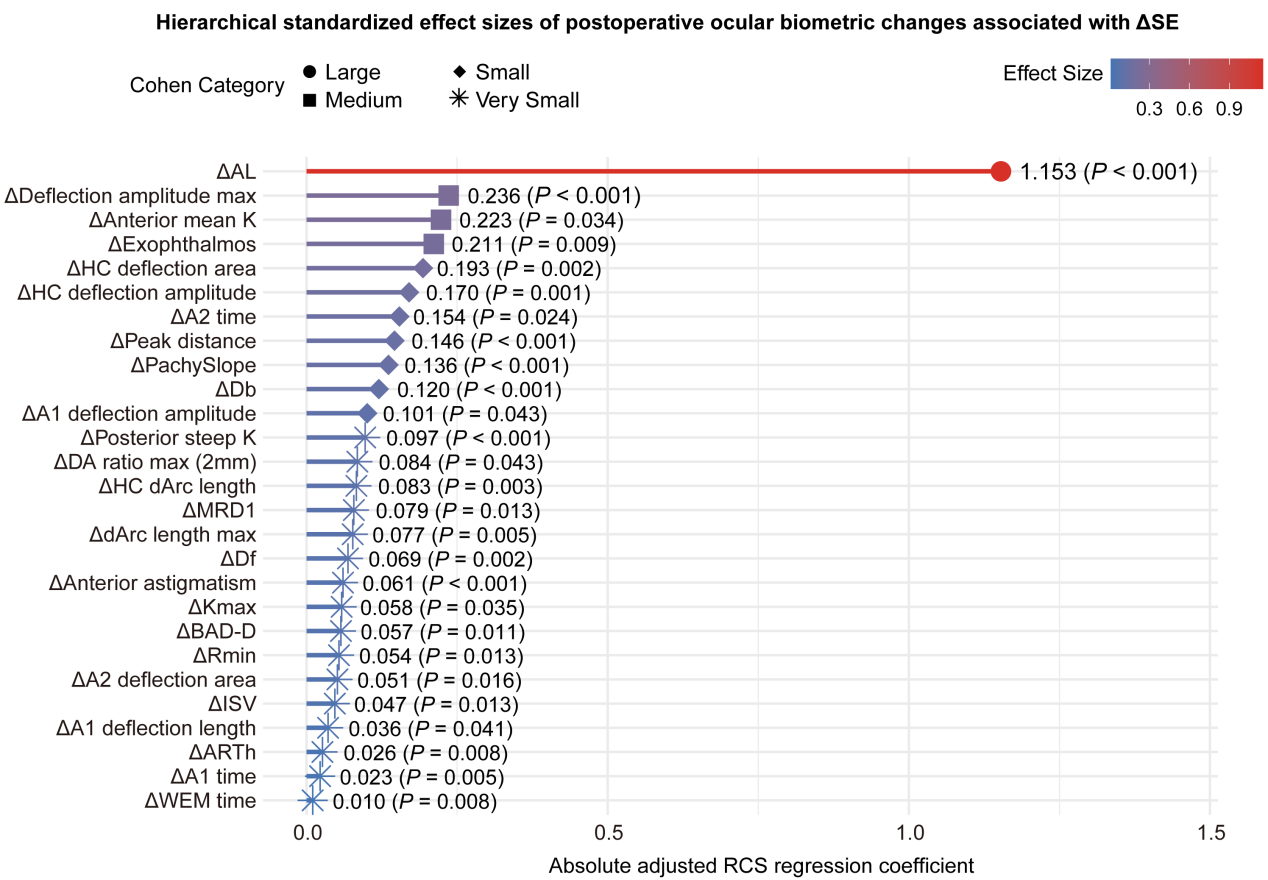


**Supplementary Analysis S4. Investigation of exposure–response relationships between baseline ocular biometric parameters and postoperative refractive change**

**Supplementary Methods S4**

To further explore exposure–response relationships between baseline ocular biometric parameters and postoperative ΔSE, non-standardized multivariable GEE models were fitted with a Gaussian family and identity link. Each baseline parameter was examined in a separate model to reduce multicollinearity, with adjustment for age, sex, baseline ocular parameters (AL, CCT, bIOP, CAS, exophthalmos), thyroid function indices, postoperative follow-up time and orbital fat removal volume. Baseline DS and DC were excluded as exposures to avoid algebraic redundancy with SE. All other model specifications followed those described in Supplementary Methods S2.

**Supplementary Results S4**

Baseline ocular biometric parameters exhibited heterogeneous patterns of nonlinear associations with postoperative ΔSE, including monotonic, U-shaped, and inverted U-shaped patterns. Complete results are presented in Additional File 11, and significant associations are depicted in Additional File 12.

Among clinical parameters, baseline exophthalmos showed a monotonic positive nonlinear association with ΔSE (*P* = 0.002), indicating that greater proptosis was associated with smaller myopic shifts or larger hyperopic shifts. In contrast, baseline AL showed a monotonic negative nonlinear association (*P* = 0.003), with longer AL associated with greater myopic shifts or smaller hyperopic shifts.

Among corneal biomechanical parameters, integrated indices including SP-A1 (*P* = 0.004) and CBI (*P* = 0.011) exhibited significant inverted U-shaped associations with ΔSE, indicating that intermediate biomechanical stiffness levels correspond to minimal myopic or maximal hyperopic shift, whereas both lower and higher extremes are associated with increased myopic or reduced hyperopic shift. In addition, bIOP (*P* = 0.032) showed a monotonic negative association with ΔSE, whereas SSI (*P* = 0.034) showed a monotonic positive association, indicating differential contributions of intraocular pressure–related stiffness properties to postoperative refractive change.

Among corneal morphological variables, baseline IHA demonstrated a significant U-shaped association with ΔSE (*P* = 0.026), indicating that intermediate levels of corneal asymmetry correspond to maximal myopic shift, whereas both lower and higher extremes are associated with reduced myopic or increased hyperopic shift. Corneal volume within the 10-mm zone, reflecting global corneal structural integrity and biomechanical stability, also showed a significant U-shaped association with ΔSE (*P* < 0.001).

Across these nonlinear associations, a consistent pattern was observed, in which larger myopic shifts tended to cluster around regions corresponding to the troughs of U-shaped curves and the extremes of inverted U-shaped curves. Based on this cross-parameter convergence, we propose the term Refractive Susceptibility Zone (RSZ) as a descriptive construct to summarize regions within which baseline ocular biometric parameters were associated with relatively greater postoperative myopic shift following orbital decompression, potentially reflecting heterogeneous biomechanical responses to postoperative structural alterations.

Importantly, RSZ should not be interpreted as a discrete anatomical or physiological entity, but rather as an empirical pattern derived from nonlinear exposure–response relationships observed in this cohort. This conceptualization may provide a useful framework for describing heterogeneous sensitivity of refractive outcomes to baseline ocular biometric states following orbital decompression. However, given its exploratory nature, RSZ requires external validation and should be interpreted cautiously in other populations and surgical contexts.

**Supplementary Analysis S5. Sensitivity analysis of baseline ocular biometric predictors of clinically significant myopic drift excluding thyroid function indices**

**Supplementary Methods S5**

To assess the robustness of the primary findings, sensitivity analyses were performed by excluding thyroid function indices (FT3, FT4, and TSH) from the covariate adjustment set using the same multivariable logistic GEE framework. All model specifications and multicollinearity assessment were identical to the primary analysis.

**Supplementary Results S5**

Overall, patterns of associations remained largely consistent with the primary analyses. Complete results are presented in Additional File 13, and significant associations are depicted in Supplementary Fig. S5.

Several baseline parameters were associated with a lower risk of clinically significant myopic drift. Among corneal biomechanical parameters, a more gradual corneal thickness profile (higher ARTh: OR = 0.622, *P* = 0.002) and longer first applanation time (A1 time: OR = 0.752, *P* = 0.047) were independently associated with reduced risk. Corneal morphological parameters, including a flatter posterior corneal surface (higher [i.e., less negative] posterior flat K; OR = 0.776, *P* = 0.034) and a larger corneal volume within the 10-mm zone (larger CV; OR = 0.759, *P* = 0.045) also conferred decreased risk of clinically significant myopic drift.

Conversely, several baseline parameters were associated with a higher risk of clinically significant myopic drift. Specifically, corneal biomechanical parameters, including longer WEM time (OR = 1.341, *P* = 0.011) and higher PachySlope (OR = 1.426, *P* = 0.030) were independently associated with increased risk. Corneal morphological variables, including a steeper anterior corneal surface (higher anterior steep K: OR = 1.803, *P* = 0.002; higher anterior mean K: OR = 1.537, *P* = 0.018) and greater anterior corneal astigmatism (OR = 1.483, *P* = 0.010) were also correlated with elevated risk. Additionally, greater deviation in corneal thickness distribution (higher Dt: OR = 1.458, *P* < 0.001; higher Dp: OR = 1.398, *P* = 0.013) and greater internal anterior chamber depth (internal ACD: OR = 1.386, *P* = 0.012) also conferred greater risk of clinically significant myopic drift.

Multicollinearity diagnostics continued to indicate no meaningful collinearity among predictors (all VIFs < 5), supporting the numerical stability of the sensitivity models.

Overall, sensitivity analysis excluding thyroid function indices yielded highly consistent results compared with the primary analysis. The direction of associations remained unchanged across all major predictors, and no effect reversals were observed. Although minor variations in effect sizes were noted for several variables, the overall pattern of significant predictors remained stable, confirming the robustness of the primary findings.

**Supplementary Fig. S5. Sensitivity analysis of associations between baseline ocular biometric parameters and clinically significant myopic drift.** Forest plot of adjusted ORs with 95% CI from multivariable logistic GEE models. Models were adjusted for age, sex, baseline ocular parameters (AL, CCT, bIOP, CAS, exophthalmos), postoperative follow-up time and orbital fat removal volume. ORs represent the change in odds of clinically significant myopic drift per one standard deviation increase in the corresponding baseline parameter. Each row represents one baseline parameter analyzed in an individual model. Points indicate adjusted ORs, and horizontal bars denote 95% CI. *P* values correspond to Wald tests for the parameter of interest within each model. AL, axial length; ARTh, Ambrósio’s relational thickness (horizontal); CI, confidence interval; CV, corneal volume; Dp, pachymetric progression index deviation; Dt, thinnest point deviation; GEE, generalized estimating equations; Internal ACD, internal anterior chamber depth; K, keratometry; Max VIF, maximum variance inflation factor; OR, odds ratio; PachySlope, pachymetric slope; WEM time, maximum whole eye movement time


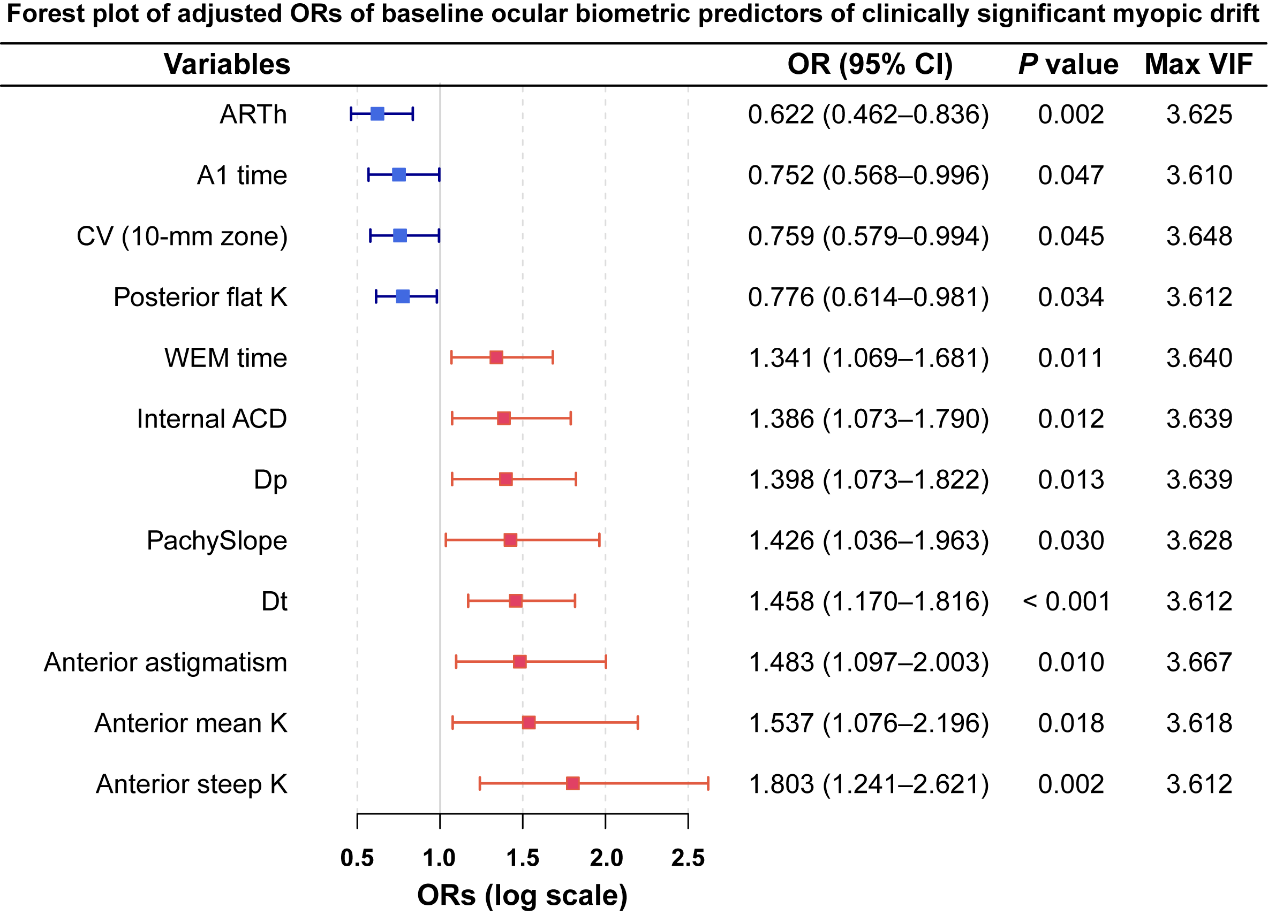


**Supplementary Analysis S6. Sample size adequacy assessment and contextual considerations**

Given the retrospective nature of this study, no formal a priori sample size calculation was performed. The sample size was determined by the availability of eligible patients with complete longitudinal data during the study period.

To provide contextual reference, approximate sample size considerations were explored using G*Power 3.1 under simplified assumptions (medium effect size f = 0.25, α = 0.05, power = 0.80, assumed within-subject correlation = 0.5), yielding an estimated requirement of approximately 28 independent units under a simplified repeated-measures design as implemented in G*Power 3.1.

Within the context of the present study, this value is provided solely as a heuristic benchmark. It is not directly applicable to the clustered data structure of the present GEE analyses, in which statistical inference is driven primarily by the number of independent patient-level clusters (n = 46), rather than by repeated measurements or bilateral eye-level observations.

Therefore, this benchmark is reported for descriptive and contextual interpretation only and should not be interpreted as a formal power or sample size estimation for the primary or secondary GEE-based analyses.

No formal sample size estimation was performed for any exploratory or secondary analyses, including subgroup comparisons, binary outcomes of clinically significant postoperative myopic drift, baseline predictor analyses, or RCS-based exposure–response modeling.
