# Supplementary figures and images for: Longitudinal refractive changes following orbital decompression in thyroid eye disease: dominant role of axial configuration and globe position with contributions from surgical technique and corneal biomechanics
Source: Eye Vis (Lond). 2026 Jul 22;13:32. doi: 10.1186/s40662-026-00499-9 (PMC13390221; doi:10.1186/s40662-026-00499-9)

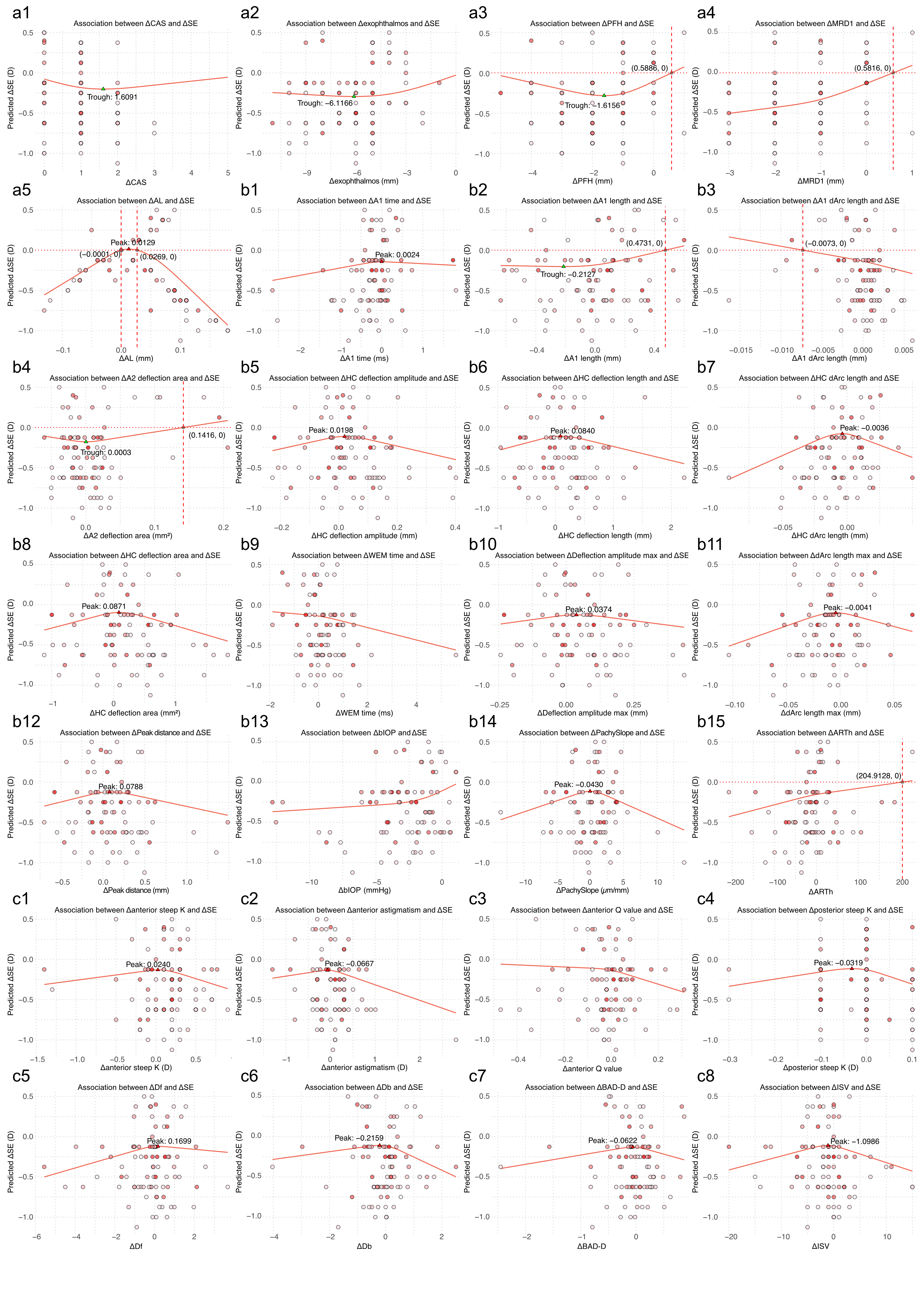

Supplement: Supplementary file 8 — Additional file 8. Significant exposure–response associations between postoperative ocular biometric changes and postoperative ΔSE identified from non-standardized RCS-based GEE models. Exposure–response curves for postoperative changes in ocular biometric parameters that showed statistically significant associations with postoperative ΔSE in non-standardized RCS-based GEE models. For clarity of interpretation, all parameters are grouped into three predefined categories according to their clinical and biomechanical characteristics: Group A: clinical variables encompassing orbital structural parameters, inflammatory activity, eyelid position metrics, and AL; Group B: Corvis ST parameters representing corneal biomechanical responses; Group C: corneal morphological variables describing corneal shape and tomographic features. For each panel, associations were further classified according to their functional forms, and key features such as extremum points, were indicated where applicable. AL, axial length; ARTh, Ambrósio’s relational thickness; BAD-D, Belin/Ambrósio Display D index; bIOP, biomechanically corrected intraocular pressure; CAS, Clinical Activity Score; dArc, deformation arc; Db, back elevation deviation; Df, front elevation deviation; GEE, generalized estimating equations; HC, highest concavity; ISV, index of surface variance; MRD1, margin reflex distance 1; PFH, palpebral fissure height; RCS, restricted cubic splines; WEM time, maximum whole eye movement time [file 40662_2026_499_MOESM8_ESM.png]

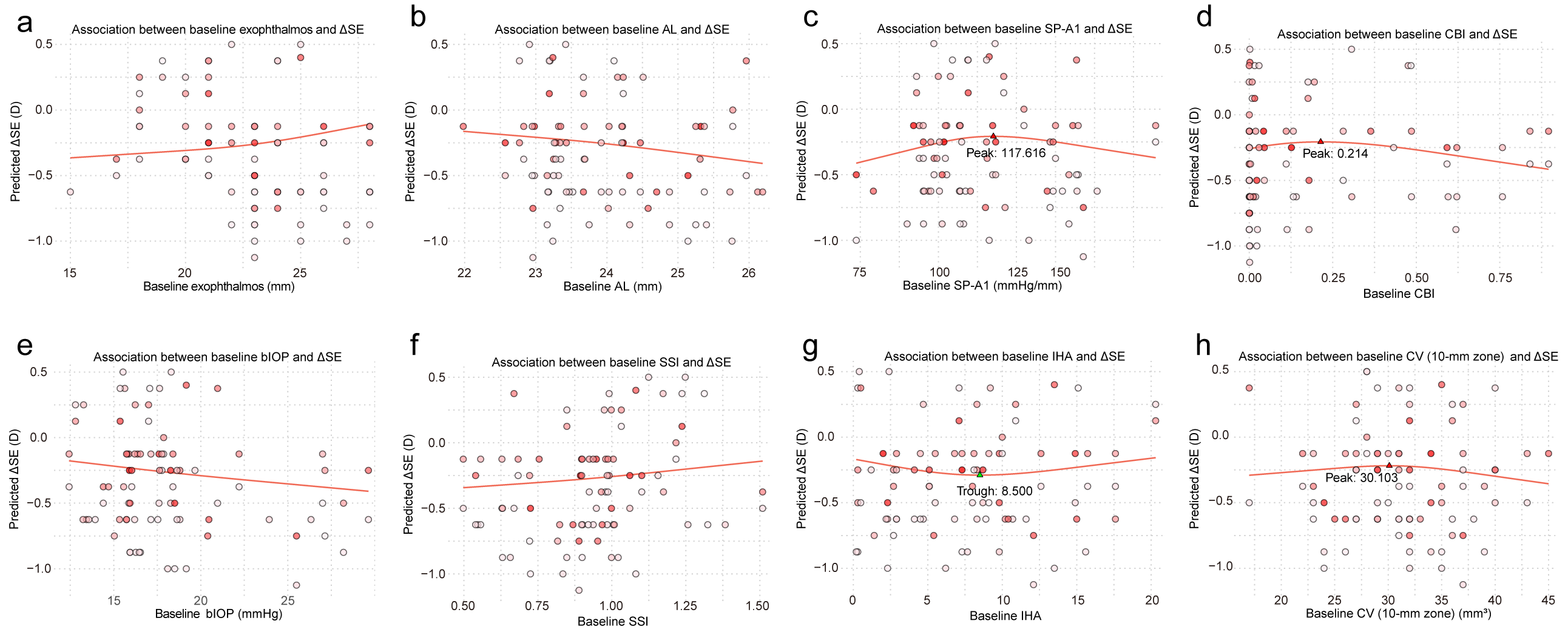

Supplement: Supplementary file 12 — Additional file 12. Significant exposure–response associations between baseline ocular biometric parameters and postoperative ΔSE identified from non-standardized RCS-based GEE models. Exposure–response curves for baseline ocular biometric parameters that demonstrated statistically significant associations with postoperative ΔSE in non-standardized RCS-based GEE models. Associations are categorized according to their functional patterns, and key features such as extremum points are indicated where applicable. GEE, generalized estimating equations; RCS, restricted cubic splines; SE, spherical equivalent [file 40662_2026_499_MOESM12_ESM.png]
